# Potentially Toxic Elements and Natural Radioactivity in Nasser Lake Sediments: Environmental Risks in a Key Egyptian Freshwater Lake

**DOI:** 10.3390/toxics13090745

**Published:** 2025-08-31

**Authors:** Esraa S. El-Shlemy, Ahmed Gad, Mohammed G. El Feky, Abdel-Moneim A. Mahmoud, Omnia El-Sayed, Neveen S. Abed

**Affiliations:** 1 Department of Biological and Geological Sciences, Faculty of Education, Ain Shams University, Cairo 11341, Egypt; 2 Geosciences Department, College of Science, United Arab Emirates University, Al Ain 15551, United Arab Emirates; 3 Geology Department, Faculty of Science, Ain Shams University, Cairo 11566, Egypt; 4 Geochemical Exploration Department, Nuclear Materials Authority, El-Maadi, Cairo P.O. Box 530, Egypt

**Keywords:** Inland Lakes, mineralogy, potentially toxic elements, sediment pollution, ecological risk, radiation hazards, Nasser Lake, Egypt

## Abstract

A necessary evaluation of freshwater ecosystem pollution levels and radiation risks remains crucial for maintaining environmental health, especially within economically developing areas. This study presents a comprehensive evaluation of the mineralogical, geochemical, and radiological characteristics of sediments in Nasser Lake, Egypt, to determine potential ecological and health risks. Forty sediment samples were collected from multiple locations, including both surface and bottom sediments, for analysis of textural attributes, mineral composition, potentially toxic elements, and natural radionuclides (^238^U, ^232^Th, and ^40^K). Results revealed sand-dominated sediments with low organic matter content. The heavy mineral assemblages derived from Nile River inputs, wind-deposited materials, and eroded igneous and metamorphic rocks. Geochemical analysis showed that arsenic, cadmium, chromium, and lead concentrations exceeded upper continental crust background values, with enrichment factors and geo-accumulation indices indicating significant anthropogenic contributions. The pollution indices revealed heavy contamination levels and extreme ecological risks, which were primarily driven by arsenic and cadmium concentrations. Radiological assessments detected activity concentrations of ^238^U, ^232^Th, and ^40^K below the world average, with hazard indices indicating minimal radiological risk except where localized hotspots were present. The study emphasizes the need for targeted monitoring and sustainable management practices to mitigate pollution and preserve the crucial freshwater environment of Nasser Lake.

## 1. Introduction

Freshwater ecosystems such as rivers, lakes, and wetlands provide essential benefits for environmental sustainability, economic development, and human health [1,2]. However, the water and sediment components within these vital ecosystems are increasingly polluted due to anthropogenic activities and natural processes. The main sources of contamination include industrial discharge, urban wastewater, agricultural runoff, atmospheric deposition, and natural weathering processes [1,3].

Lake sediments are highly complex and dynamic systems that act as both sinks and secondary sources for various pollutants, including persistent organic compounds, polycyclic aromatic hydrocarbons (PAHs), potentially toxic elements (PTEs), and naturally occurring radioactive materials (NORMs) [4,5]. Due to their high adsorption capacity, lake sediments can reduce elevated concentrations of contaminants such as PTEs and NORMs in the overlying water column [6,7]. However, gradual changes in sediment dynamics and chemistry, driven by shifting environmental conditions, can release weakly bound contaminants into the water through diffusion, advection, and sediment resuspension. This process leads to secondary contamination, which negatively impacts the health and function of aquatic ecosystems [7,8,9]. Accurate identification of specific contaminants in sediments and associated waters is essential, as some of these substances pose serious threats to human health and the environment [6].

Priority PTEs such as arsenic, cadmium, chromium, nickel, and lead receive special attention due to their persistence and bioaccumulating toxic effects [10,11,12]. Long-term exposure to these metals can lead to severe health issues, including cancer, birth defects, kidney failure, and neurological damage [13,14]. Anthropogenic activities are the primary sources of PTEs, accounting for 75% of these pollutants [1,3]. This is equally true for NORMs, which include uranium, thorium, and their decay products. NORMs accumulate in sediments before entering the food chain and water sources, leading to internal radiation exposure [15,16]. Long-term exposure to NORMs increases the risk of kidney, lung, and bone cancers, as well as other chronic health conditions and radiological damage [17,18]. Although NORMs in sediments generally pose a limited radioactive risk, elevated concentrations in some locations require additional monitoring [19].

In recent decades, geochemical and radiological assessments have become essential for determining contaminant loads in freshwater lakes, particularly in regions experiencing rapid land-use changes and upstream industrial activity [20,21,22]. Mineralogical analysis provides a precise understanding of sediment composition, revealing the effectiveness of contaminant binding and enabling researchers to assess the movement and environmental fate of these pollutants [23,24].

Niu et al. [25] analyzed heavy metal concentrations in surface sediments from Taihu Lake, China. Their results revealed that Cd was the primary contributor to ecological risks due to moderate to heavy pollution levels, while Pb had the greatest impact on total sediment toxicity. Industrial sources significantly affected sediment quality, contributing to 64.9% of Taihu Lake’s heavy metal content. Aziman et al. [26] conducted a comprehensive radiological survey and evaluated heavy metal contamination in Chini Lake, Malaysia. Their measurements showed that annual effective dose values in lake sediments exceeded 1 mSv/y, raising safety concerns for both the lake ecosystem and human health. Baguma et al. [13] assessed heavy metal pollution levels in sediments from Port Bell, Lake Victoria, Uganda. A health risk assessment indicated that dredging activities pose modest non-carcinogenic risks despite contamination, though immediate health dangers remain low. Cetin et al. [6] evaluated potentially toxic elements (PTEs) and natural radioactivity levels in water and sediments from Boraboy Lake, Turkey, for environmental pollution assessment. Their findings suggested minimal health risks, with heavy metal and radiation measurements remaining within internationally permissible limits. Szarłowicz et al. [21] investigated sediment contamination in Rybnik Lake, Poland. The study detected varying concentrations of radionuclides and heavy metals, with ^40^K reaching the highest levels and copper showing significant contamination.

Nasser Lake is a large freshwater reservoir located in southern Egypt, connected to the Aswan High Dam. The lake plays an essential role in agricultural support, national water security, and biodiversity protection. However, its sediments are increasingly contaminated due to industrial activities, agricultural runoff, and natural erosion processes. Most previous studies on Nasser Lake have focused on sedimentation [27,28], sediment transportation [29,30], and remote sensing and GIS applications [31,32]. In contrast, the geochemical and radiological characteristics of the lake’s sediments have received less research attention [33,34,35]. Moreover, most of these studies were either site-specific, confined to certain classes of contaminants, or did not include an integrated evaluation of ecological and human health risks. Hence, there is a clear research gap in providing a comprehensive characterization of Nasser Lake sediments in terms of their mineralogical, geochemical, and radiological characteristics and in relating these to potential ecological and radiological risks. The present study aims to fill this gap. This study integrates mineralogical, geochemical, and radiological analyses of Nasser Lake sediments to assess pollution levels, evaluate radiation hazards, identify potential sources, and evaluate presumed ecological and health risks. The findings will support informed decision-making for the sustainable management of one of Egypt’s most vital freshwater resources.

## 2. Materials and Methods

### 2.1. The Study Area

**Figure 1:**
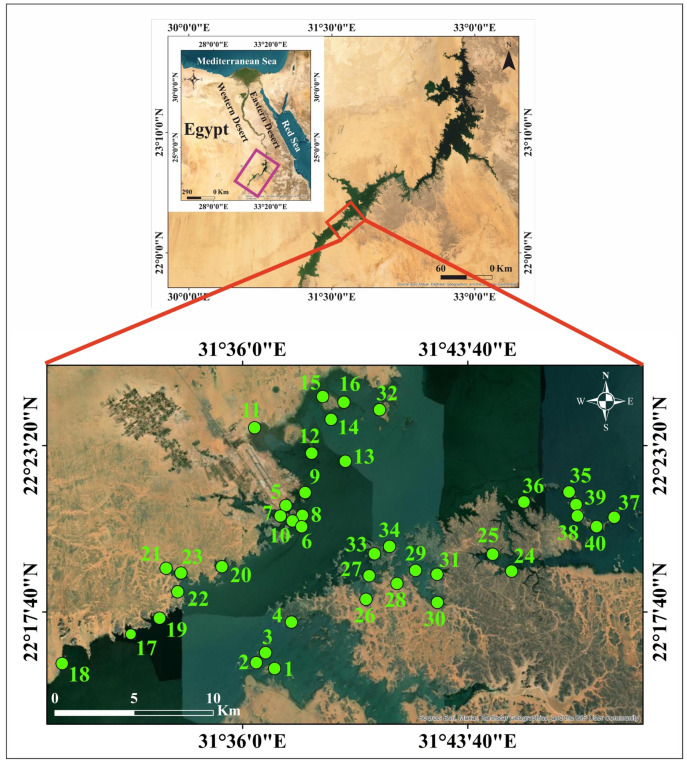
The study area and sampling locations.

High Dam Lake is located between 20°27′–23°58′ N and 30°07′–33°15′ E, at an elevation of 182 m above mean sea level in the lower Nile River Basin, near the Egyptian border with Sudan. It stretches for 500 km in length, with 350 km along Egypt’s border (where it is called Nasser Lake) and 150 km along Sudan’s border (where it is known as Nubia Lake). The lake was formed in the late 1960s and early 1970s as a result of the construction of the Aswan High Dam, which was built approximately 5 km south of Aswan city, upstream of the old Aswan Dam [35,36]. Nasser Lake is one of the world’s longest man-made lakes, supplying nearly 95% of Egypt’s freshwater [34]. It is considered Egypt’s primary water reservoir. Due to the country’s growing tourism, industrial, and agricultural sectors, it has become essential to monitor and assess any changes in its water quality. The study area extends for about 403 km^2^ in the southern part of Nasser Lake in Egypt. It is bounded by longitudes 31°29′–31°49′ E and latitudes 22°15′–22°26′ N (Figure 1). Southern Egypt is an arid region, with extremely hot summers and warm winters. According to a geological analysis of the lake region, the most commonly exposed rock units surrounding the lake are the Nubian Sandstone Formation. Additionally, sand dunes and sand sheets are present there [31]. The geology of the western area of the lake is dominated by Cretaceous to Quaternary sedimentary successions, with exposures of metamorphic and igneous rocks from the Late Precambrian basement, as well as Phanerozoic foreland volcanics from the Late Cretaceous and Mid-Tertiary ages [37]. The eastern side of the lake is primarily composed of foreland deposits (the Nubian Sandstone Formation) that are underlain by Precambrian metamorphic and igneous rocks. These rocks outcrop at various sites in the area and are unconformably overlain by the foreland deposits, which are represented by the Cretaceous Nubian Sandstone Formation and Quaternary deposits that fill the wadis, their tributaries, and the wide plains between highlands. The geomorphologic features of the lake area mainly include embayments (khors), headlands and peninsulas, islands, and the Nubian Delta [38].

### 2.2. Sample Collection and Preparation

Forty sediment samples were collected from the eastern and western banks of southern Nasser Lake, including khors and islands (Figure 1). Thirty-two samples represent surface sediments, while the remaining eight represent bottom sediments. Hand-picking was used to collect samples from depths of less than one meter, whereas a simple grab sampler was used for depths greater than one meter. The collected samples were stored in polyethylene bags and transported to the laboratory within a few hours in ice tanks. The samples were air-dried before employing the coning and quartering procedure to prepare representative samples for different analyses.

### 2.3. Samples Analyses

#### 2.3.1. Sediment Granulometry

The organic matter content (OM%) in the studied sediments was estimated using the loss-on-ignition method [39], where two grams of sample were ignited in a pre-weighed platinum crucible at 550 °C for 2 h in a muffle furnace. Grain-size distribution was analyzed using wet and dry sieving techniques. For wet sieving, a 4 Ø (63 µm) mesh was used to separate gravel and sand fractions from the mud fraction in 50 g sediment samples, following Lewis and McConchie [40]. The gravel and sand fractions were then dry-sieved using −1, 0, 1, 2, 3, and 4 Ø standard mesh sieves in a Ro-Tap shaker for 20 min.

#### 2.3.2. Mineralogy

Following the recommendations of Mange and Maurer [41], bromoform (with a specific gravity of 2.85 g/cm^3^) was used as the heavy liquid to separate heavy minerals from the fine and very fine sand-size fractions of the sediment samples. After washing the separated heavy fraction with ethyl alcohol, Canada balsam was used to mount the heavy mineral grains on slides. The percentages of heavy minerals in each sample were determined by counting at least 300 grains per sample in each heavy mineral mount. The “ribbon counting” method [41] was employed to identify and count the heavy mineral grains under an Olympus polarized microscope. Following Hubert [42], the ZTR maturity index, which represents the percentage of zircon, tourmaline, and rutile relative to the non-opaque, non-micaceous heavy minerals, was calculated. A Prisma E environmental scanning electron microscope (ESEM) equipped with an EDX analyzer was used to examine heavy minerals and determine their chemical compositions in the mud, fine sand, and very fine sand fractions of representative samples.

#### 2.3.3. Geochemical Analysis

The major and trace element analyses were conducted using an Axios Sequential Wavelength Dispersive X-Ray Fluorescence (WD-XRF) Spectrometer (PANalytical, 2021, Malvern Panalytical, Malvern, UK) following the ASTM E-1621 [43] standard guide for elemental analysis by wavelength-dispersive X-ray fluorescence spectrometry. Major element data were obtained by fusing samples in an electric furnace with a 1:10 ratio of sample (1 g) to flux agent (10 g), which consisted of a mixture of 66% lithium tetraborate and 34% lithium metaborate. The spectrum was scanned and processed using Omnian/WROXI software’s mathematical algorithms. Trace elements were measured by pressing 6 g of sample with 1.5 g of binder wax using an automated HERZOG pressing machine (HERZOG, Osnabrück, Germany). The pressed disk spectrum was scanned and processed using PRO-TRACE calibration standards. To ensure the accuracy and precision of the XRF analyses, all operating parameters of the WD-XRF were software-controlled. The instrument was calibrated using certified standard reference materials: Ball Clay (BCS-CRM No. 348; Bureau of Analyzed Samples Ltd., Middlesbrough, UK) and Green River Shale (SGR-1; United States Geological Survey). Analytical accuracy was routinely checked by analyzing these standards alongside the samples. Replicate analyses of selected samples yielded relative standard deviations of less than 5%.

#### 2.3.4. Radiometric Analysis

The lake sediment samples were dried, homogenized, placed into 100 mL standard plastic containers, and tightly sealed with Teflon tape around the screw necks and wide vinyl tape over the caps. They were stored for 30 days before the examination. The radiogenic gases ^220^Rn and ^222^Rn were restricted from escaping due to the ingrowth from the decay of U and Th, allowing secular equilibrium to occur between ^238^U, ^232^Th, and their daughter products [44]. The activity concentrations of ^238^U (via ^234^Th at 0.0633 MeV), ^232^Th (via ^212^Pb at 0.2386 MeV), and ^40^K (1.461 MeV) in the sediment samples were measured using a well-calibrated sodium iodide scintillation detector (76 mm × 76 mm NaI(Tl)). The detector, housed in an aluminum case, was coupled to a photomultiplier tube and shielded from external radiation by a lead-brick container (5 cm thick) with a lead cover. A copper cylinder (0.6 cm thick) further protects the system from induced X-rays. The detector’s energy calibration was performed using standard point sources (^60^Co and ^137^Cs). Each sample was counted for 1000 s.

### 2.4. Environmental Assessment Indices

#### 2.4.1. Metals Contamination Indices

Single pollution indices, such as the Enrichment Factor (EF) [45] and the Geo-Accumulation Index (I_geo_) [46,47], were used to assess the anthropogenic contributions of each toxic element exceeding its natural levels in the sediments of the study area. Additionally, to perform an integrative assessment of sediment quality and evaluate the potential ecological risks caused by the potentially toxic element (PTE) contamination, while accounting for multi-element pollution, integrated pollution indices such as the Improved Nemerow’s Pollution Index (P_n_) [48], Pollution Load Index (PLI) [49,50], and Potential Ecological Risk Index (PERI) [49] were determined.

Background values often serve as a baseline for accurately differentiating between elevated concentrations of chemical elements caused by anthropogenic activities and their natural concentrations. The sediments covering the study area were derived from the weathering of various rock types. Thus, the background values were based on the chemical composition of the Upper Continental Crust (UCC) [51]. A summary of the single and integrated pollution index equations and categories is provided in Table S1 (Supplementary Materials).

#### 2.4.2. Radiation Hazard Indices Calculation

To assess the external radiation hazards from the activity concentration of the measured radionuclides in the studied sediment of Nasser Lake, the following indices were calculated: the radium equivalent activity index (Ra_eq_) [52,53], the external hazard index (H_ex_) [53,54], the absorbed dose rate (D) [53], the annual effective dose (AEDE) [53], and the excess lifetime cancer risk (ELCR) [55,56,57]. Definitions and equations used to calculate these radiation hazard indices are provided in Table S2.

### 2.5. Data Treatment

ArcGIS (version 10.8.1; 2020) with a raster interpolation technique (Spline-Tension) was used to display the measured PTEs’ locations and spatial distribution maps in Nasser Lake. OriginLab (version OriginPro 2021) was used to present descriptive statistics, box-plot figures, and multivariate statistical analyses. Microsoft Excel (version Microsoft Office 365 16.0.15028.20160) was used to calculate contamination levels and health risk assessments. The Positive Matrix Factorization (PMF 5.0) model, provided by the U.S. Environmental Protection Agency (EPA), was employed to identify and quantify PTE sources.

## 3. Results and Discussion

### 3.1. Textural Characteristics

The results of the grain size analysis and the identified grain size parameters for the studied sediment samples are provided in the Supplementary Materials (Tables S3 and S4). The sediments consist predominantly of sand, with percentages ranging from 88.10% to 99.94%, and only three samples are classified as muddy sand. The grain size distribution of Nasser Lake’s sediments is influenced by sediment depth, as the clay fraction increases with depth, while the sand fraction decreases [58,59]. The high sand content in the lake khors is likely due to detrital input from nearby sand sheets and wind-drifted rocks. Current and wave activity along the shoreline contribute to additional erosion and sediment deposition. Wind action may also introduce some materials, particularly sand [59].

Based on the cumulative curves (Figure S1) and the data presented in the boxplots of mean size (Mz), sorting coefficient (σ_I_), skewness (SK_I_), and kurtosis (KG) (Figure S2), the studied sediments consist primarily of fine sands with some medium sand and are mostly moderately to moderately well sorted. Skewness varies but tends to be nearly symmetrical to finely skewed, while kurtosis ranges from platykurtic to very leptokurtic, with mesokurtic and leptokurtic types being most common. These characteristics indicate texturally mature sediments [27].

The organic matter content (OM%) of these sediments ranges from 0.00 to 1.99%, averaging 0.85%. Sediment texture is considered the primary control on organic matter distribution in the lake [58,60]. The sandy texture and low organic content suggest deposition in a high-energy environment.

### 3.2. Mineralogy and Mineral Chemistry

**Figure 2:**
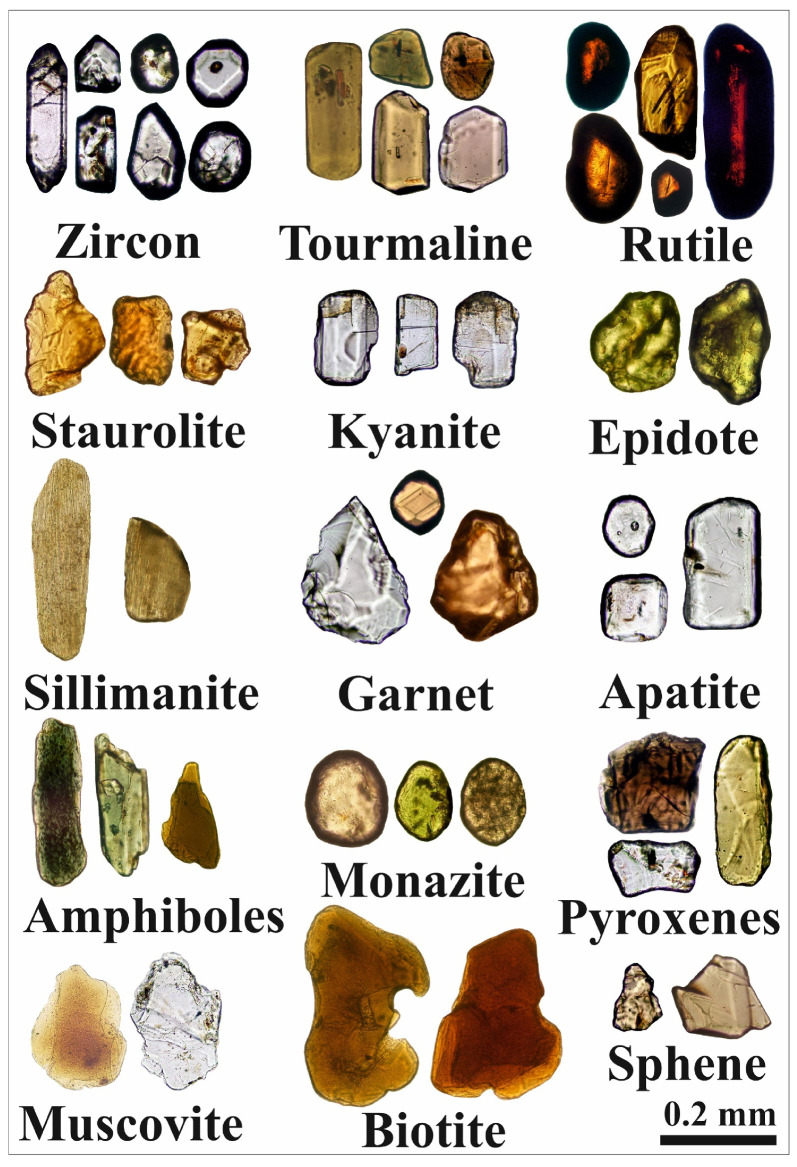
Photomicrographs of the non-opaque heavy minerals’ assemblage in Nasser Lake sediments.

The majority of the light fraction of minerals in the study area consists of quartz and feldspar grains. The heavy mineral index percentages in the studied sediments range from 0.16% to 30.37%, with an average of 4.27% (Table S5). The heavy minerals in the very fine and fine sand-sized fractions include both opaque and non-opaque types. Opaque heavy minerals dominate the heavy mineral assemblages in the investigated sediments, comprising between 21.15 and 81.64% (average 57.51%) (Table S5). Iron oxide minerals, such as magnetite, hematite, ilmenite, and leucoxene, are the most common opaque minerals in the sediments of the study area [59,61]. Titanomagnetite minerals were also detected. The non-opaque minerals include amphiboles, pyroxenes, zircon, tourmaline, rutile, garnet, epidote, monazite, kyanite, sphene, staurolite, apatite, sillimanite, biotite, and muscovite in varying proportions (Figure 2). These minerals can be categorized as ultrastable, metastable, and unstable [41].

**Figure 3:**
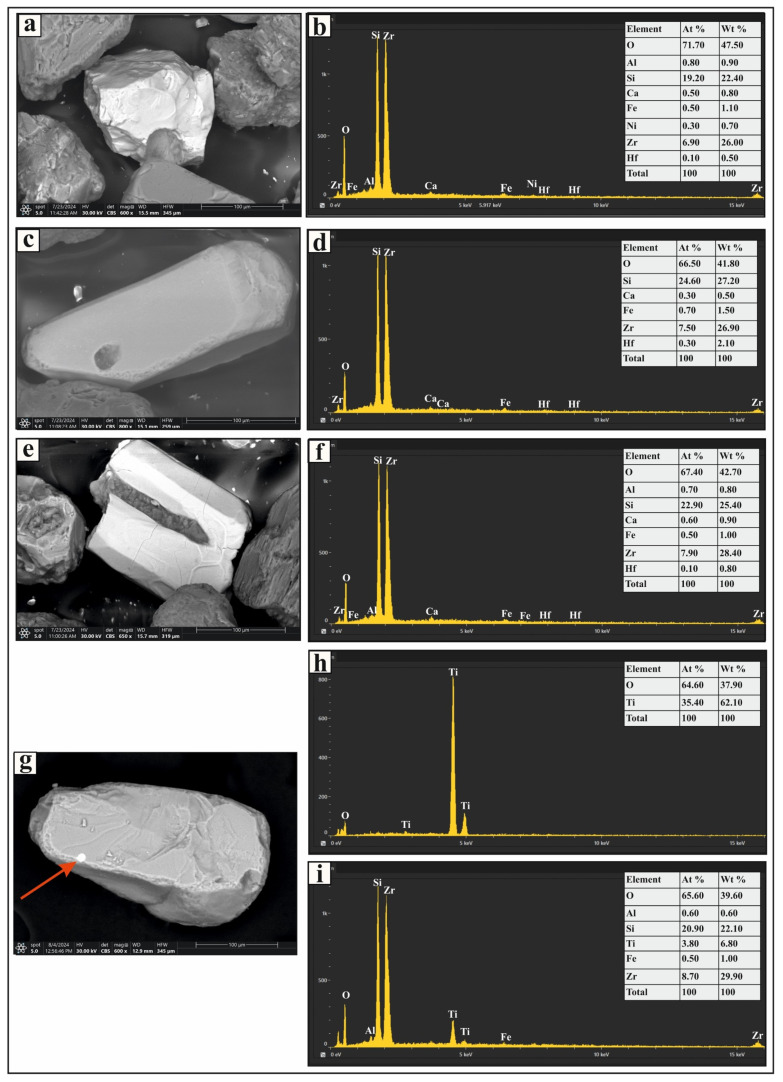
SEM micrographs and EDS analysis results of selected heavy minerals separated from the very fine and fine sand-sized fractions of Nasser Lake sediments: (**a**,**c**,**e**) zircon grains with various morphologies; (**b**,**d**,**f**) display the EDS spectra for the zircon grains; (**g**) a rutile grain with a zircon inclusion; (**h**,**i**) EDS spectra for the rutile grain and the zircon inclusion, respectively.

**Figure 4:**
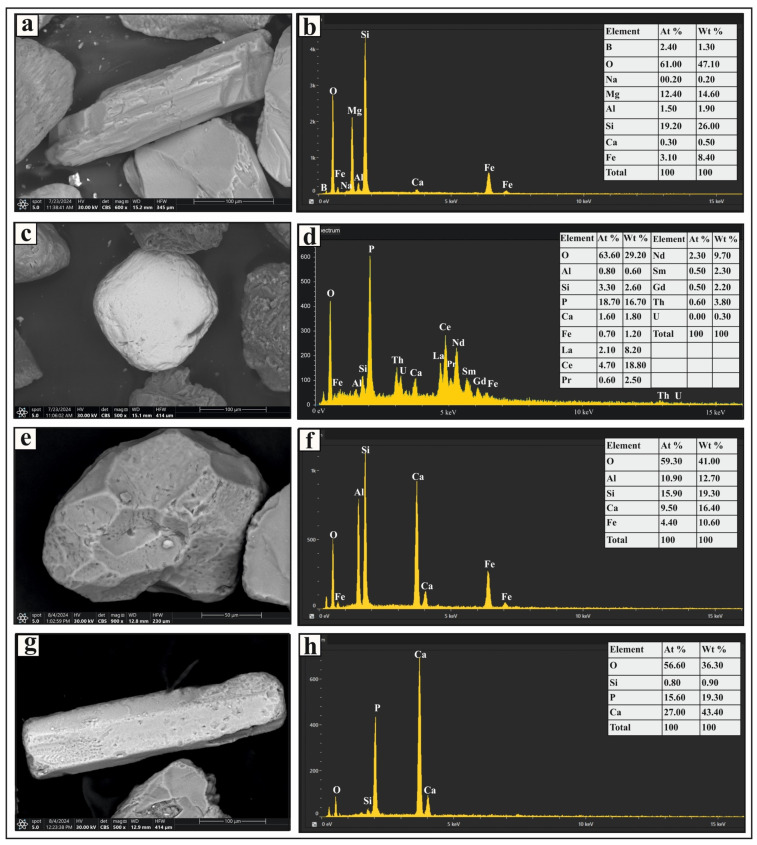
SEM micrographs and EDS analysis results of selected heavy minerals separated from the very fine and fine sand-sized fractions in Nasser Lake sediments: (**a**,**b**) show a prismatic tourmaline grain and its EDS spectrum, respectively; (**c**) shows a subrounded monazite grain; (**d**) shows the EDS spectrum for the monazite grain, including radioactive and rare earth elements; (**e**,**f**) present a euhedral garnet grain with a pitted surface and its EDS spectrum, respectively; and (**g**,**h**) display a prismatic apatite grain and its EDS spectrum, respectively.

The ultrastable group includes zircon, rutile, and tourmaline (Figure 2). The most prevalent mineral in this category is zircon, which appears as euhedral, bipyramidal, prismatic, rounded, and subrounded crystals (Figure 3a,c). It is colorless, although some grains contain inclusions and fractures (Figure 3e). Rutile occurs as prismatic, oval, and subrounded grains with amber, brownish-red, and deep-red hues. These grains exhibit significant pleochroism and thick black borders, and some contain zircon inclusions (Figure 3g). Tourmaline grains in the studied sediments are prismatic (Figure 4a), elongated, and subrounded, displaying green and brown colors with strong pleochroism. Inclusions are common within these grains.

The metastable group includes monazite, garnet, epidote, staurolite, kyanite, sillimanite, and apatite (Figure 2). Monazite grains are pale yellow to greenish yellow in color, with rounded, subrounded, and oval forms. Some grains exhibit brownish stains and surface pitting. Trace amounts of uranium, thorium, and rare-earth elements were detected in certain grains (Figure 4c). Garnet occurs as colorless, pinkish, or pinkish-brown grains with euhedral, rhombohedral, and subangular shapes, some displaying pitted surfaces (Figure 4e). Epidote grains are subangular to subrounded and exhibit lemon-yellow or yellowish-green hues. Staurolite grains are bright yellow to golden yellow, with irregular or subangular shapes; some contain inclusions. Kyanite grains are colorless, prismatic, and cleavable, often with rounded corners. Sillimanite grains appear as either short, stout prismatic fragments or long, slender prisms. The fibrous variety (fibrolite) consists of fine, subparallel to parallel, needle-like crystals, which are colorless or exhibit light green to light brown hues. Apatite grains are colorless and vary in shape, including euhedral short prismatic, long slender prismatic (Figure 4g), hexagonal, subrounded, and oval forms. Some display smooth curved terminations, surface pitting, or inclusions.

**Figure 5:**
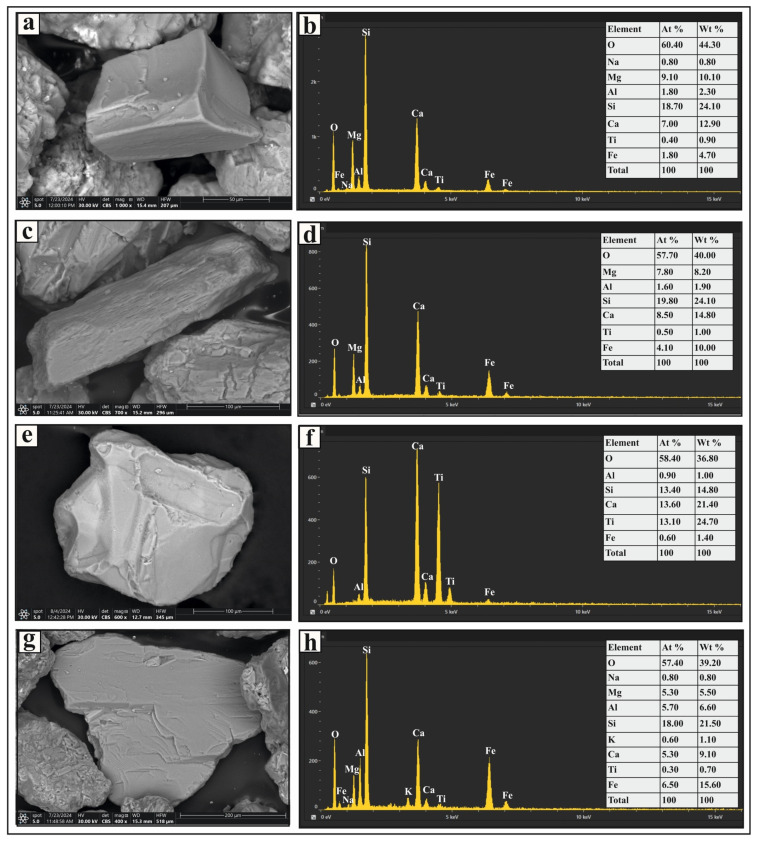
SEM micrographs and EDS analysis results of selected heavy minerals separated from the very fine and fine sand-size fractions in the studied sediments: (**a**,**b**) show a short prismatic pyroxene grain and its EDS spectrum, respectively; (**c**,**d**) present a prismatic amphibole grain with distinct cleavage and its EDS spectrum, respectively; (**e**,**f**) depict a sphene grain and its EDS spectrum, respectively; and (**g**,**h**) display a platy biotite grain and its EDS spectrum, respectively.

The unstable group includes pyroxenes, amphiboles, sphene, biotite, and muscovite (Figure 2). Pyroxene grains in the studied sediments are green, brown, or colorless, with elongated, short-prismatic (Figure 5a), and irregular shapes. Some grains contain fractures and inclusions, with augite being the predominant pyroxene mineral. Amphibole grains are mostly composed of thin flakes, displaying prismatic and irregular shapes in varying shades of brown and green. They exhibit strong pleochroism and distinct cleavage planes (Figure 5c), with hornblende as the most abundant amphibole. Sphene grains are subangular to irregular (Figure 5e), with honey-yellow and light-brown colors and a resinous luster. Biotite grains are irregular, platy, and brown or reddish brown, primarily composed of thin flakes (Figure 5g); some grains contain inclusions. Muscovite grains are colorless or yellow-stained, with a platy, irregular form. Their outlines range from rounded to irregular, and some also contain inclusions.

The ZTR index provides a quantitative measure of the mineralogical maturity of heavy mineral assemblages [42]. An increase in the ZTR index reflects higher concentrations of zircon, tourmaline, and rutile, while concentrations of other non-opaque heavy minerals decrease. Mineralogically mature sediments exhibit a significantly higher ZTR index. In the investigated sediments (Table S5), the ZTR index ranges from 1.84% to 59.77% (average: 22.42%). Since the ZTR index is below 75%, the studied sediments are considered mineralogically immature [62]. This conclusion is further supported by the relatively high percentage of unstable heavy minerals (pyroxenes and amphiboles) in the sediments [63].

The heavy mineral assemblage and the presence of both prismatic and rounded forms of ultrastable heavy minerals (zircon, rutile, and tourmaline) in the studied sediments suggest that the study area has a distinct heavy mineral source. Kandil et al. [59] proposed some of these sources, concluding that they may include the River Nile, which primarily supplies sediments and reworked rocks that are derived from upstream mountainous erosion. Additionally, wind and drainage systems contribute sediments eroded from various exposed sources, such as igneous and metamorphic rocks, sandstone, and sand accumulations, located along the western and eastern banks of Nasser Lake. These sediments are characterized by their low maturity. Another potential source is eroded material from exposed beaches and coastal cliffs. Large quantities of sediment in southern Egypt were derived from the complex wadi system dissecting the Red Sea Hills [64].

The majority of the investigated sediments contain considerable amounts of pyroxenes and amphiboles, indicating a contribution from the basement rocks of the Red Sea Hills to the distribution of these minerals. The presence of staurolite, kyanite, epidote, biotite, muscovite, and garnet suggests a metamorphic origin [65]. The River Nile sediments are derived from various rock types. While magnetite, ilmenite, hornblende, augite, zircon, tourmaline, monazite, and apatite point to both mafic and silicic igneous sources, staurolite, sillimanite, kyanite, garnet, and epidote indicate a metamorphic origin for a portion of the sediments [23,66].

Scanning electron microscope (SEM) analysis of the mud-sized fraction revealed several minerals, including precious metals such as gold (Figure S3a) and silver (Figure S3c,e). This fraction also contains radioactive and rare-earth element (REE)-bearing minerals, such as monazite (Figure S4a), brannerite (Figure S4c), allanite (Figure S4e), thorite (Figure S4g), uranophane (Figure S5a), and cerianite (Figure S5c), as well as other silicate (Figure S5e) and carbonate (Figure S5g) minerals enriched in rare-earth elements.

### 3.3. Pollution Assessment

#### 3.3.1. PTEs Distribution

**Figure 6:**
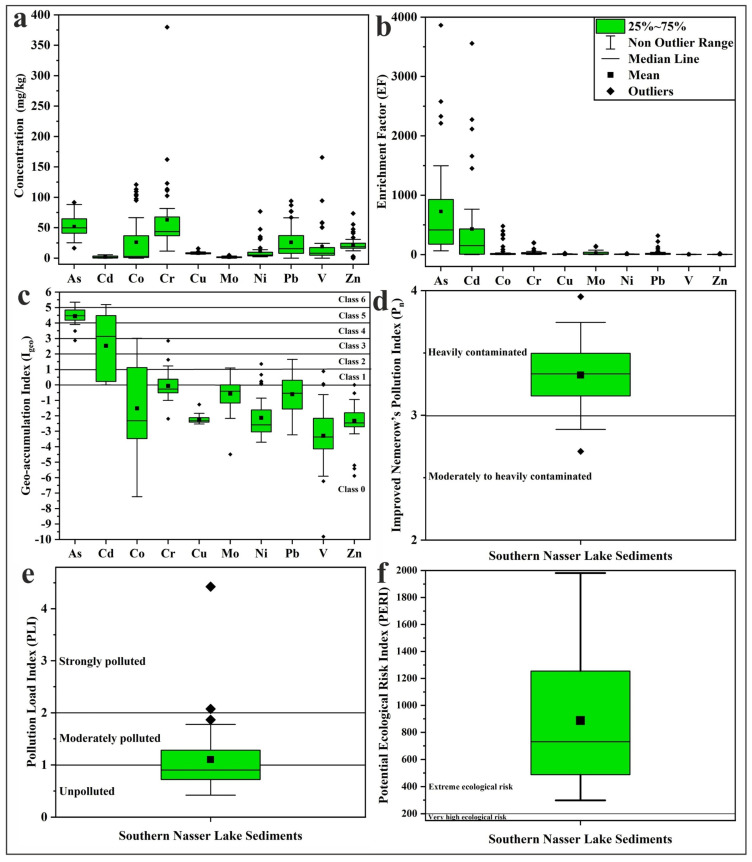
Boxplots of (**a**) PTE concentrations, (**b**) EF, (**c**) I_geo_, (**d**) P_n_, (**e**) PLI, and (**f**) PERI values in Nasser Lake sediments.

The elemental composition obtained by XRF analysis of the studied sediment samples (Table S6) indicates that Nasser Lake sediments exhibited high silica content (average: 87.61%) and relatively low Al_2_O_3_ content (average: 1.82%), which is attributed to the low mud percentage in these samples. The concentrations of the measured PTEs in the sediments are presented in Table 1 and Figure 6. The highest PTE concentrations were recorded for Cr and As, while Mo and Cd showed the lowest values. Generally, the concentrations of As, Cd, Co, Cr, Cu, Mo, Ni, Pb, V, and Zn ranged from 16.50 to 91.60, BDL to 5.40, BDL to 120.80, 11.50 to 379.70, 6.50 to 15.60, BDL to 4.80, 2.30 to 76.80, BDL to 93.90, BDL to 165.50, and BDL to 73.50 (mg/kg), respectively. The average concentrations of these elements follow the order Cr (63.07 mg/kg) > As (51.80 mg/kg) > Co (26.05 mg/kg) > Pb (25.96 mg/kg) > Zn (21.30 mg/kg) > V (18.85 mg/kg) > Ni (11.30 mg/kg) > Cu (8.05 mg/kg) > Cd (1.67 mg/kg) > Mo (1.55 mg/kg).

Compared to the background values of the Upper Continental Crust (UCC) [51] (Table 1), the concentrations of As, Cd, Co, Cr, Cu, Mo, Ni, Pb, V, and Zn exceeded these values in 100.0%, 75.0%, 35.0%, 77.5%, 0.0%, 40.0%, 15.0%, 40.0%, 5.0%, and 2.5% of the studied samples, respectively. This suggests that anthropogenic activities may have influenced the distribution of PTEs to varying degrees. Although there are no direct sources of heavy metal pollution entering Lake Nasser, it receives anthropogenic contaminants from fishing boats, cruise ships, sewage from fishermen, and waste from other anthropogenic activities discharged into the Sudanese Main Nile, Blue Nile, and White Nile. These contaminants include industrial, agricultural, domestic, and mining waste [33,67,68].

#### 3.3.2. Metal Contamination and Ecological Risk

The EF values were calculated to assess the impact of anthropogenic activities on the PTEs in the studied sediment samples, and the results are presented in Table 2 and Figure 6. All analyzed samples showed very high EF values (>40) for As, indicating extremely high enrichment [45], and more than half of the samples also exhibited very high EF values for Cd. Cr, Ni, Cu, Mo, and Pb showed significant enrichment (EF = 5–20) in a considerable number of samples, while Zn and Co displayed the same level of enrichment in some samples. Zn exhibited moderate enrichment (EF = 2–5) in 50% of the samples. The combination of As, Cd, Pb, and Zn suggests a mixed source from industrial and transportation activities [69]. V showed no enrichment (EF < 2) in more than 50% of the studied sediments. The high degree of enrichment of As and Cd in the Nasser Lake sediments can be most plausibly attributed to anthropogenic sources. Arsenic is commonly associated with the use of arsenic-containing pesticides and industrial effluents, while Cd is usually derived from phosphate fertilizers, mine tailings, and combustion processes [70,71].

The calculated I_geo_ values in the study area (Table 2; Figure 6) indicated that the sediments were heavily to extremely contaminated with As in the majority of samples, with I_geo_ values ranging from 4 to 5 (Class 5) [47]. The I_geo_ values for Cd covered a wide range (Class 1 to Class 6), and a considerable portion of the samples fell into the same class of contamination as As. In contrast, most I_geo_ values for the other measured metals were in Class 0 (<0), indicating practically uncontaminated sediments. For Co, a significant number of samples had I_geo_ values in Classes 1, 2, and 3, reflecting a range of pollution levels. Some samples exhibited I_geo_ values for Cr in Class 1 (0–1), suggesting uncontaminated to moderately contaminated sediments for this element.

The present results are generally consistent with earlier studies on Lake Nasser sediments [33,34,35], although some differences were observed. For instance, Darwish [33] reported lower average concentrations of Cr and Pb, while Rizk et al. [35] found lower average concentrations of Cd and Pb compared to our findings. Furthermore, Imam et al. [34] observed relatively low anthropogenic enrichment for Cd using I_geo_ and EF, whereas our results indicate higher values. These discrepancies could suggest an increasing anthropogenic influence in recent years.

The EF and I_geo_ values from the present study can be contextualized by comparing them with results from other freshwater lakes. For instance, Niu et al. [25] reported comparable I_geo_ values for Cr, Cu, Ni, Pb, and Zn in China’s Taihu Lake, but they found moderate to heavy Cd pollution alongside lower As values. Similarly, Aziman et al. [26] observed high EF and I_geo_ values for As and Pb in Malaysia’s Chini Lake. In contrast, Szarłowicz et al. [21] reported higher I_geo_ values for Cu and Ni in Poland’s Rybnik Lake. Relative to these systems, the EF and I_geo_ values for As and Cd in Lake Nasser are exceedingly high, ranking it among the most heavily affected lakes. This highlights a critical need for continued monitoring of contaminant inputs into the lake.

However, EF and I_geo_ are single-element indices and do not adequately represent overall contamination status from all PTEs at each sampling location. To properly assess pollution levels across the study area, comprehensive multi-element indices such as the P_n_, PLI, and PERI are necessary [48,72].

**Figure 7:**
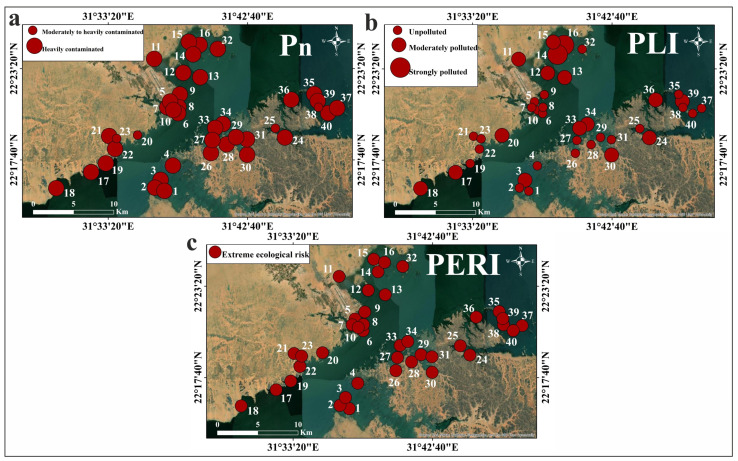
Spatial distribution maps of the values of the multi-element indices in the Nasser Lake sediments: (**a**) P_n_; (**b**) PLI; (**c**) PERI.

The calculated P_n_ values of the sediments are presented in Table 3 and Figure 6. Most samples exhibited P_n_ values ranging from 3 to 4, indicating heavy contamination [48]. The contamination factor-based PLI values (Table 3; Figure 6) for most sediments ranged from <1 to 1–2, representing unpolluted to moderately polluted conditions [49,50]. All calculated PERI values indicated an extreme ecological risk (>200) [48,49], as shown in Table 3 and Figure 6. The spatial distribution maps of Nasser Lake sediment Pn, PLI, and PERI are presented in Figure 7. These maps clearly visualize spatial variation of these indices (P_n_, PLI, and PERI) across the study area, providing valuable assistance to decision-makers in identifying contaminated or high-risk zones.

**Figure 8:**
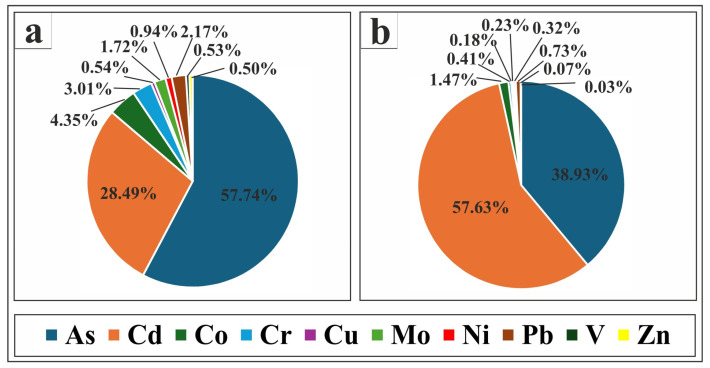
Pie charts showing the percentages of each PTE contribution to certain pollution index values in Nasser Lake sediments: (**a**) shows the PTEs’ contribution percentages to PLI values; (**b**) displays the PTEs’ contribution percentages to PERI values.

The contribution of each PTE to the PLI and PERI values in the study area is presented in Figure 8. The figure shows that As contributed the most to PLI values (57.74%), followed by Cd (28.49%). In contrast, Co, Cr, Pb, and Mo had lower contributions (4.35%, 3.01%, 2.17%, and 1.72%, respectively), while Ni, Cu, V, and Zn contributed very little (0.94%, 0.54%, 0.53%, and 0.50%, respectively). For PERI values, Cd was the dominant contributor (57.63%), followed by As (38.93%). Co’s contribution was low (1.47%), whereas Pb, Cr, Ni, Mo, Cu, V, and Zn exhibited minimal contributions (0.73%, 0.41%, 0.32%, 0.23%, 0.18%, 0.07%, and 0.03%, respectively).

#### 3.3.3. PTEs Source Apportionment

Correlation analysis is an efficient way to assess the intensity and linkage between the analyzed PTEs through Pearson correlation coefficients (PCC). PTEs with significant correlations may originate from the same source [73]. Correlation values of 0.00–0.19, 0.20–0.39, 0.40–0.59, 0.60–0.79, and 0.80–1.00 can be considered indicative of very weak, weak, moderate, strong, and very strong correlations, respectively [74]. The PCC analysis of Lake Nasser sediments suggests potential origins of PTEs (Table 4). The moderate to very strong positive correlation among Cr, Cu, Ni, V, Zn, and major oxides such as Fe_2_O_3_, Al_2_O_3_, and TiO_2_ (r = 0.51–0.89) suggests that the PTEs mainly belong to the detrital fraction and are largely derived from natural geogenic sources, such as the weathering of upstream rocks and soils. These elements are intimately associated with Fe and Al oxides, as they are co-precipitated or adsorbed on oxide minerals during sedimentation. The strong to very strong relationship of these elements with TiO_2_, an immobile and conservative oxide, supports a lithogenic origin rather than an anthropogenic input [5,72]. The moderate to strong correlations between organic matter and Cu (r = 0.48), Ni (r = 0.57), V (r = 0.55), and Zn (r = 0.65) may indicate the contribution of organic complexes in the transport and retention of these metals. The negative correlation between most of the measured metals and the percentage of mud content suggests the absence of an influence from the mud fraction on their distribution. This finding agrees with that of Farhat and Aly [68], who stated that the distribution of heavy metals in Lake Nasser sediments is mainly controlled by organic matter rather than the mud fraction. The very low, consistently weak, or even negative correlations of As, Cd, Co, and Pb, along with the distinct negative trend of Mo with almost all variables, suggest different sources, which are likely anthropogenic.

**Figure 9:**
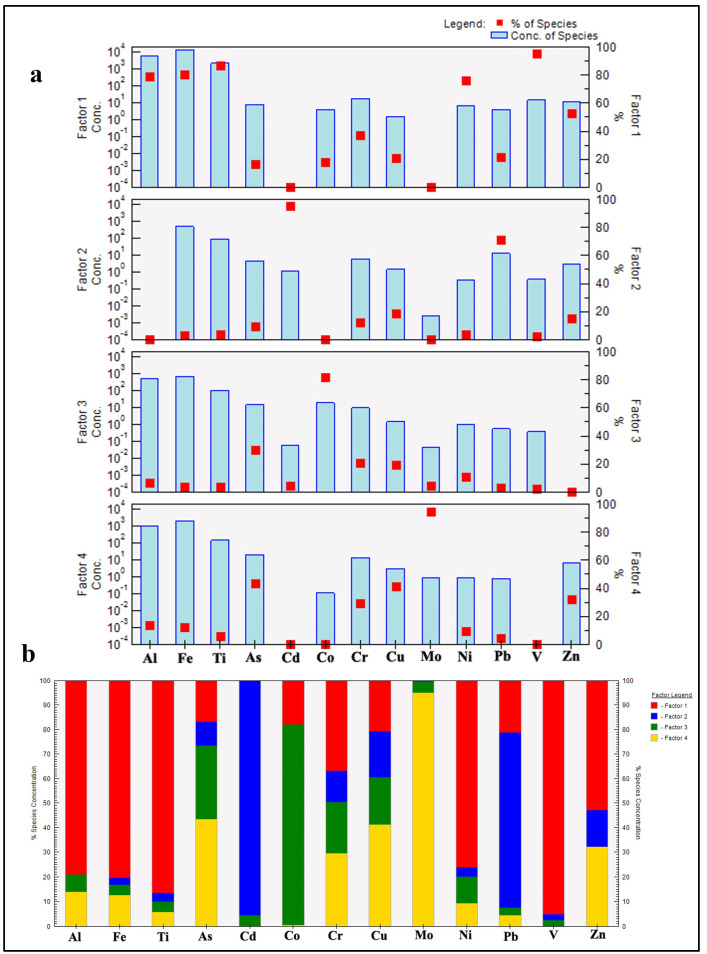
PMF model. (**a**) Factor profiles; (**b**) factor contributions.

The Positive Matrix Factorization (PMF) model was used to identify and quantify the contribution of each source to PTEs. The model identified four distinct sources contributing to the observed PTE concentrations in the sediments of Nasser Lake (Figure 9). Factor 1 is dominated by high loadings of Al (79%), Fe (80%), and Ti (86.5%), which are well-established tracers of natural geological processes such as the weathering of parent rocks and soil erosion [75]. The significant contributions of V (95%), Ni (76.2%), and Zn (53%) further support a geogenic origin, as these elements are commonly enriched in mafic and ultramafic rocks prevalent in the Nile River basin [66]. The moderate contributions of Zn, Cr, Cu, Pb, and As suggest that, while these elements may have anthropogenic inputs, their presence is also strongly influenced by natural sedimentary processes [76]. This aligns with the PCC results, where Cr, Cu, Ni, and Zn exhibited moderate to very strong correlations with Al_2_O_3_, Fe_2_O_3_, and TiO_2_, reinforcing their lithogenic association. Thus, this factor can be attributed to a natural contribution of PTEs from rock weathering and lithogenic input into the lake environment.

Factor 2 is characterized by high contributions from Cd (95.4%) and Pb (71.1%), all of which are indicative of industrial activities and fossil fuel combustion. Cd, in particular, exceeded the Upper Continental Crust (UCC) values in 75% of the samples and exhibited extremely high EF and Igeo indices, underscoring its dominance in this pollution group. Cd is commonly linked to industrial activities and phosphate fertilizers [77], while Pb emissions are historically associated with leaded gasoline [78]. The moderate contributions of Zn, Cr, and As suggest additional mixed sources, such as metal smelting and urban runoff [79]. These results align with the elevated EF and I_geo_ values for Cd and Pb, indicating significant anthropogenic enrichment. Thus, this factor can be attributed to fossil fuel combustion and industrial emissions of these PTEs.

Factor 3 shows high loadings of As (30.1%), Co (81.7%), Cr (20.8%), and Cu (19.3%). These elements are commonly linked to agrochemical inputs and urban effluents, including arsenic-containing pesticides, fertilizers, and domestic wastewater. A significant positive correlation was observed between As and Co (R = 0.75; Table 4). The association between As and Co may be indicative of a common anthropogenic source, such as agrochemicals, or wastewater effluents that can be co-contaminated with As and Co [71,80]. Although Cr and Cu are partly lithogenic, they are also associated with industrial materials and urban emissions [81,82]. These findings are consistent with the moderate enrichment of these metals observed in many samples, as well as their ecological risk profiles. This factor suggests that agricultural and urban activities have contributed to the PTE pollution.

Factor 4 is uniquely dominated by Mo (95%), suggesting atmospheric input through the dry deposition of Mo-rich aerosols, likely from remote industrial activities [83]. Mo is used in alloys, lubricants, and catalysts and can also be a by-product of tungsten and copper mining. Other anthropogenic sources of Mo include petrochemical plants and fossil fuel combustion [84]. Additionally, As (43.3%) and Cu (41.3%) are significantly represented, aligning with emissions from ore smelting and high-temperature industrial processes [85]. Moderate contributions from Zn and Cr further suggest atmospheric fallout, possibly from electroplating, metallurgical, or vehicular sources. This factor can be attributed to atmospheric deposition.

The PMF results corroborate the pollution assessment in Section 3.3.2, where As and Cd were identified as the primary contributors to ecological risks (PERI > 200). The dominance of Cd in Factor 2 and As in Factors 3 and 4 underscores their anthropogenic origins, consistent with their high EF (>40) and I_geo_ (Class 5–6) values. Conversely, the geogenic association of Cr, Ni, and Zn in Factor 1 aligns with their lower contamination levels in single-element indices. Notably, there are no major industrial activities in the study area, aside from small fishing and tourism ports; thus, industrial contributions are likely linked to distant sources along the River Nile Basin. The Nile and its tributaries drain extensive upstream catchments in Sudan, Ethiopia, and beyond, transporting suspended sediments and dissolved loads. These loads receive inputs from multiple anthropogenic activities, including industrial discharges, mining, agricultural runoff, and urban wastewater. Consequently, PTE contamination in the river’s sediments has emerged as a pressing environmental concern, with potential implications for human health throughout the Nile Basin. This region, spanning 11 countries in East and Central Africa, requires a comprehensive understanding of PTE pollution to devise effective mitigation and management strategies. Although research has historically focused on Egypt, growing evidence suggests that upstream countries, including Ethiopia, Uganda, Kenya, and Tanzania, also face significant risks from PTEs in sediments [86,87,88,89].

Biological activity and biomineralization processes can also influence the redistribution of PTEs in lake sediments, affecting contaminant mobility and bioavailability. Microorganisms and benthic organisms may contribute to the transformation of PTEs through redox processes, biosorption, and the formation of biogenic minerals, consequently potentially decreasing or increasing pollution risk. However, the role of these biological processes in Nasser Lake’s sediments has not been thoroughly investigated. Future research should investigate the effects of biomineralization and microbial activity on PTE dynamics to gain a better understanding of their environmental fate and ecological implications.

### 3.4. Radiometry of the Studied Sediments

#### 3.4.1. Activity Concentrations

**Figure 10:**
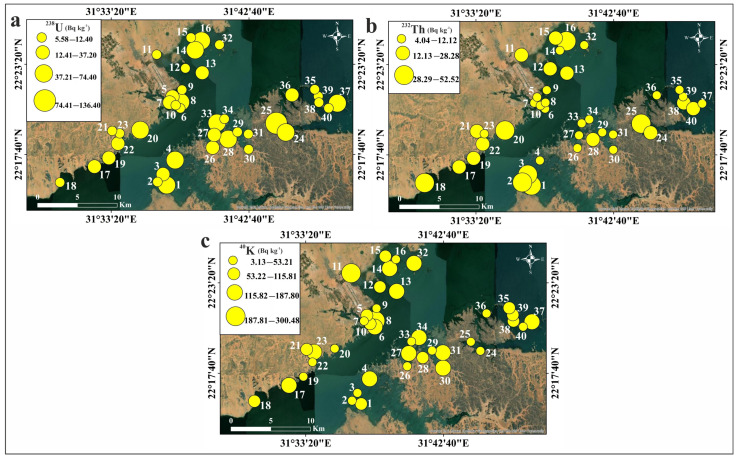
Spatial distribution maps of the natural radionuclides in Nasser Lake sediments: (**a**) ^238^U; (**b**) ^232^Th; (**c**) ^40^K.

The activity concentrations of ^238^U, ^232^Th, ^226^Ra, and ^40^K in the studied sediments are displayed in Table 5. The average values of these concentrations are 30.94, 17.83, 14.47, and 95.34 Bq kg^−1^ for ^238^U, ^232^Th, ^226^Ra, and ^40^K, respectively. The permissible levels are 33, 45, 32, and 412 Bq kg^−1^ for ^238^U, ^232^Th, ^226^Ra, and ^40^K, respectively [56]. All the measured activity concentrations for different radionuclides are not far from what was reported by Kandil et al. [59] and El Azab et al. [61] and are lower than the permissible levels, suggesting their safe use. The results indicate that the measured ^40^K concentration significantly exceeds those of both ^238^U and ^232^Th concentrations. This suggests that, in general, the radioactive element ^40^K is prevalent in the studied sediments. Compared to U and Th, potassium is more abundant in magmatic rocks and is a major component of many rock-forming minerals [90,91]. The current investigation revealed that ^238^U, ^232^Th, and ^40^K levels in Nasser Lake sediments are of an entirely natural origin. The spatial distribution of the measured radionuclides in each sediment sample is displayed in Figure 10.

#### 3.4.2. Radionuclide Behavior and Activity Ratios

Sediments can be classified into three distinct categories based on their Th/U ratios [92]. The first type has a Th/U ratio ranging from 0.012 to 0.81. These sediments are transported from a uranium source and subsequently stabilized through repeated reworking. The second type exhibits a Th/U ratio between 1.47 and 1.49. The relatively high Th content is attributed to slightly increased scavenging of U caused by leaching-recharging processes. The third type of sediment shows a Th/U ratio between 1.49 and 5.47. These sediments experience ineffective weathering processes, leading to rapid rock fragment accumulation and the common presence of detrital radioactive minerals such as xenotime, samarskite, thorite, and euxenite in such settings.

**Figure 11:**
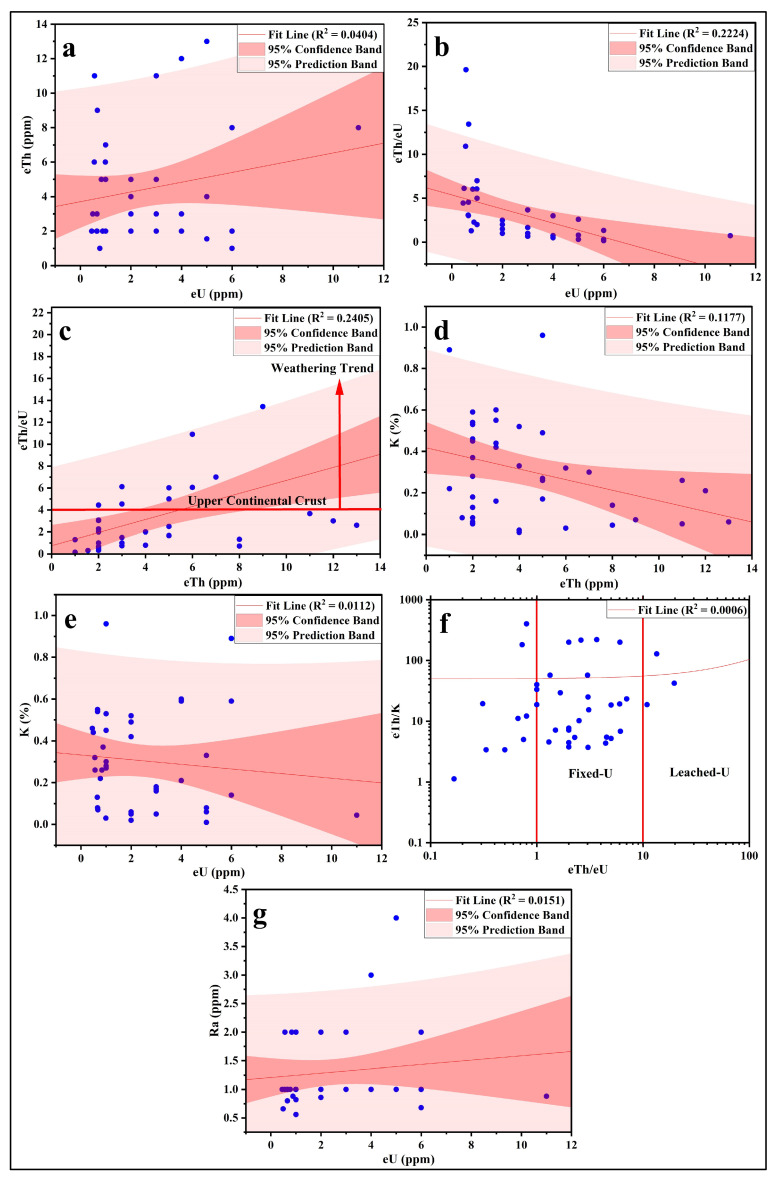
Scatter plots show the relation between the studied radionuclides (**a**) eU vs. eTh; (**b**) eU vs. eTh/eU; (**c**) eTh vs. eTh/eU; (**d**) eTh vs. K; (**e**) eU vs. K; (**f**) eTh/eU vs. eTh/K; (**g**) eU vs. Ra.

The data reveal that the eTh/eU ratios of the analyzed sediments fall within the third group, indicating limited weathering and swift deposition of rock detritus (Table 5; Figure 11a). Consequently, detrital radioactive minerals like xenotime, samarskite, thorite, and euxenite are predominant. Mineralogical analysis of the studied sediments confirms the presence of thorite, monazite, and zircon. The eTh/eU ratio varies from 0.17 to 19.64 (average: 3.38), diverging from the CHARAC (charge and radius control) value of 2.8. This suggests uranium mobilization from certain sources alongside enrichment in others. In current exogenic environments, uranium transitions into its mobile hexavalent form (U^6+^), whereas thorium remains in its immobile tetravalent form (Th^4+^). The average Th/U ratio of the studied sediments (3.38) closely aligns with that of the Upper Continental Crust [93]. However, most samples deviate, exhibiting either higher or lower ratios. This inconsistency indicates relative uptake or removal of mobile U^6+^ compared to immobile Th^4+^ under prevailing exogenic conditions. The elevated eTh/eU ratio in clay minerals (predominantly kaolin) within the karst fillings of Southern Belgium may result from preferential sorption of immobile Th onto clays, a phenomenon observed in other acidic weathering environments. Alternatively, it could reflect relative U loss, either as UO_2_^2+^ or in complex forms, compared to Th [94,95]. This hypothesis may explain the eTh/eU ratio variations in the analyzed sediments.

Radium exists only in the divalent oxidation state in solution. It is unstable and radioactive, occurring in varying concentrations across different geological materials, such as rocks, soil, and water. The four radium isotopes found in the environment ^226^Ra, ^228^Ra, ^224^Ra, and ^223^Ra) primarily originate from the natural decay series of three radionuclides: uranium (U), thorium (Th), and actinium (Ac) [96]. Radium exhibits chemical behavior similar to barium; however, its compounds are even less soluble in water and acidic solutions, whereas uranium is more easily removed compared to radium [97,98]. Consequently, weathered outcrops of radioactive ores rich in pyrite may undergo selective uranium leaching, leaving radium behind. Due to radium’s relatively high gamma activity, radiometric assays of such outcrops are significantly elevated compared to chemical assays for uranium [99].

Among radium isotopes, ^226^Ra stands out as a significant radionuclide due to its potential environmental hazards, even at low concentrations. Because of its moderate solubility in water, radium can enter groundwater through the dissolution of aquifer materials, desorption from rock or sediment surfaces, and its release from minerals during radioactive decay [100]. Notably, the radium activity concentrations in the sediments under investigation are lower than those of uranium (Table 5), suggesting that radium precipitation may be occurring in this environment due to enrichment by carbonates, sulfates, and iron oxides. ESEM analysis confirmed the presence of these complexes.

The activity ratios of ^238^U/^226^Ra increased from 0.28 to 12.50, with a mean value of 2.25 (Table 5). This increase may be associated with the fact that radium tends to be more mobile under reducing conditions [101]. The observed ^238^U/^226^Ra activity ratios can be explained by the differing geochemical properties of the two isotopes. Radium naturally exhibits intermediate mobility between U(IV) and U(VI); however, unlike uranium, it is less mobile in oxidizing environments due to its strong adsorption onto clay and iron minerals, which are frequently found in such conditions and which result from the weathering of the host rock. The mobility of radium is further reduced when high concentrations of sulfate ions suppress the mobility of ^226^Ra, forming stable (precipitated) RaSO_4_. In organic-rich soil from the Cronamuck Valley, Ireland, elevated ^238^U/^226^Ra activity ratios, reaching values as high as 9, have been documented [102].

The equilibrium factor (P) is expressed as P = eU/Ra, determined through the measurement of equivalent uranium (eU) and radium (Ra) concentrations (in ppm) using radiometric methods [103,104]. The equilibrium factor (P) deviates from unity (Figure 11g), indicating disequilibrium in the majority of samples. Studies on equilibrium factors have shown that river, groundwater, lake, spring, seawater, sediment, and soil samples exhibit distinct values [105,106].

The eTh-eU binary relation suggests no correlation between the two elements in these sediments, implying that they likely occur in different minerals or that uranium does not migrate with thorium (or vice versa) (Figure 11a). This inference is further supported by the correlations between eTh/eU, eU, and eTh (Figure 11b,c). The cross-plots of eTh-K and eU-K show no relationship between U and Th during alteration processes, likely reflecting significant variations in the redox conditions of the sampled environment. The eTh-K-eU ternary diagram indicates that uranium predominantly falls within the fixed-U sector for most samples, which may be attributed to its occurrence in refractory sediment minerals (Figure 11d–f).

The correlation coefficients of the measured radionuclides in the studied sediments (Table 6) show that ^238^U and ^232^Th are moderately positively correlated with mud content. This reveals the influence of mud on the distribution of these radionuclides, likely through adsorption [107]. As identified in Section 3.2, minerals such as monazite ((Ce,La,Th)PO_4_), uranophane (Ca(UO_2_)_2_(SiO_3_OH)_2_·5H_2_O), thorite (ThSiO_4_), and brannerite (UTi_2_O_6_) are present in the mud-sized fraction (Figures S4 and S5). The concentration of these detrital minerals in specific sampling sites, which are rich in the mud fraction, directly explains the observed radiological hotspots. ^232^Th is also moderately positively correlated with organic matter (OM), Al_2_O_3_, and Fe_2_O_3_, and strongly positively correlated with TiO_2_, which can be attributed to the scavenging nature of Fe, Mn, and Ti oxides [108]. These results are supported by the detection of radioactive minerals in the mud fraction of the investigated sediments, including monazite (Figure S4a), brannerite (Figure S4c), thorite (Figure S4g), and uranophane (Figure S5a). However, ^40^K is negatively correlated with mud, indicating that mud content does not influence its distribution. It also displays a negative correlation with the other measured radionuclides (^238^U and ^232^Th) but is positively correlated with organic matter.

#### 3.4.3. Radiation Hazard Indices

Table 5 displays the calculated values of the radiological hazard indices for the studied sediments. The radium equivalent activity index (Ra_eq_) was calculated to assess the homogeneity of the radiation dose from the measured naturally occurring radionuclides [52,53]. The Ra_eq_ values ranged between 21.97 and 120.87 Bq kg^−1^, with an average of 95.34 Bq kg^−1^. These values are significantly lower than the recommended maximum limit of 370 Bq kg^−1^ [52,53].

The external hazard index (H_ex_) for gamma radiation from naturally occurring radionuclides in the sediments ranged from 0.06 to 0.50, with an average value of 0.17. None of the H_ex_ values calculated in this study exceeded the safety level of one [54], indicating a negligible risk.

**Figure 12:**
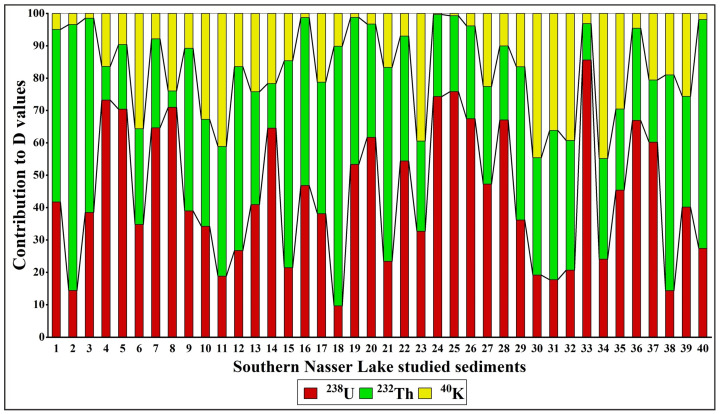
The contributions of radionuclides (^238^U, ^232^Th, and ^40^K) to the D values in Nasser Lake sediments.

The absorbed dose rate (D), which reflects an individual’s exposure to external terrestrial radiation during outdoor activities, was also evaluated. The calculated D values ranged from 9.71 to 83.11 nGy h^−1^, with an average of 29.04 nGy h^−1^. All values were lower than the worldwide average (57 nGy h^−1^; [53]), except for sample 25 (83.11 nGy h^−1^). Figure 12 illustrates the contributions of ^238^U, ^232^Th, and ^40^K to the D values in each sediment sample. The results show that ^238^U and ^232^Th contribute more significantly to the D values than ^40^K, with variations observed across the study area.

**Figure 13:**
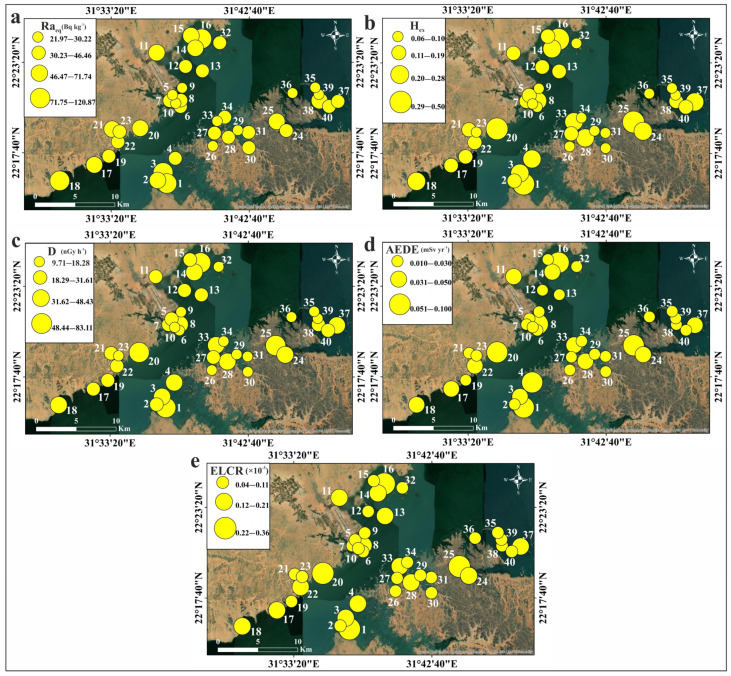
Spatial distribution maps of radiation hazard indices values in Nasser Lake sediments: (**a**) Ra_eq_; (**b**) H_ex_; (**c**) D; (**d**) AEDE; (**e**) ELCR.

The D values were used to determine the inhabitants’ annual effective doses (AEDE). The AEDE values ranged from 0.01 to 0.10 mSv yr^−1^ (average: 0.04 mSv yr^−1^). These values for all studied sediments did not exceed the world average (0.07 mSv yr^−1^; [53]), except for Sample 25, which had an AEDE value of 0.10 mSv yr^−1^. Chronic ionizing radiation exposure typically leads to additional risks, known as excess lifetime cancer risk (ELCR). ELCR factors were derived from AEDE values to better assess the health effects of external exposure to the detected natural radionuclides in Nasser Lake sediments. The calculated ELCR values ranged between 0.04 × 10^−3^ and 0.36 × 10^−3^ (average: 0.12 × 10^−3^). These values did not exceed the worldwide average (0.29 × 10^−3^; [56,57]), except for Sample 25, with a value of 0.36 × 10^−3^. This indicates that lifetime exposure to the lake’s sediments is unlikely to cause cancer. The distribution maps of the determined radiation hazard indices are shown in Figure 13. The observed radiation hotspots are due to the local sediment composition. Our mineralogical study indeed demonstrates the occurrence of uranium- and thorium-bearing mineral grains in the fine sediment fraction of the samples (Figures S4 and S5). It is likely that the hydrodynamic conditions in the lake result in the physical concentration of these dense heavy minerals in certain depositional environments, leading to the formation of local hotspots of high natural radioactivity, which need to be monitored separately from the low average risk.

## 4. Conclusions

The study provides a comprehensive assessment of the environmental status of Nasser Lake sediments. The sediment’s sandy texture with low organic content (0.85%) indicates deposition in a high-energy environment, while the heavy mineral assemblages (zircon, rutile, tourmaline) and the presence of radioactive and rare-earth-element-bearing minerals (monazite, thorite) in the fine fractions suggest complex sediment sources including Nile River inputs, aeolian deposits, and erosion of surrounding basement rocks. The high concentrations of PTEs such as and Cd with EF values over 40 and I_geo_ classes showing heavy to extreme contamination demonstrate strong anthropogenic impact. Multiple pollution assessment indices (P_n_, PLI, PERI) demonstrate heavy contamination in sediments and PERI values revealed significant ecological risk, primarily driven by As and Cd, that might harm aquatic systems and human health by bioaccumulation. The PMF model identified four contamination sources, including lithogenic, industrial/fossil fuel, agricultural/urban, and atmospheric. The natural radionuclides (^238^U, ^232^Th, and ^40^K) stayed within world averages, but certain sites displayed higher radiation levels, which require monitoring. The research demonstrates an immediate requirement to establish specific monitoring systems and construct successful sediment management practices to reduce contamination threats. Future studies must investigate seasonal changes in contaminant spread, perform in-depth source identification research, and establish remediation methods to safeguard the longevity of Nasser Lake’s vital freshwater systems and their surrounding ecosystems.

## Figures and Tables

**Table 1:** Concentrations of PTEs (mg/kg) in Nasser Lake sediments.

Sample No.	Al	As	Cd	Co	Cr	Cu	Mo	Ni	Pb	V	Zn
1	13,506	45.70	0.20	3.30	42.30	7.70	2.40	7.80	9.30	13.90	24.30
2	13,099	46.90	BDL	1.40	44.70	7.60	1.20	8.20	9.00	8.90	24.70
3	20,455	64.30	0.70	64.50	102.50	8.30	BDL	9.70	60.90	15.10	11.90
4	5451	42.90	2.30	1.90	27.40	7.40	1.30	4.20	23.10	5.20	18.40
5	3842	51.80	2.30	2.50	37.40	7.60	0.80	6.00	11.60	8.70	18.40
6	4885	42.70	3.40	2.90	27.90	7.20	1.00	2.60	56.20	3.90	16.10
7	8219	55.30	3.90	0.10	44.20	7.40	1.20	6.60	8.40	12.60	19.20
8	1217	52.90	0.70	0.90	50.70	6.80	1.50	5.00	17.40	5.00	17.10
9	1408	34.00	3.90	0.70	122.80	8.30	0.60	7.20	77.20	2.90	20.60
10	5515	41.50	2.00	2.20	43.90	7.00	2.10	5.00	22.60	8.70	20.40
11	15,877	82.40	0.20	104.70	78.60	9.80	1.30	31.30	11.10	50.30	33.80
12	19,317	56.30	0.30	14.20	70.20	9.50	0.90	32.10	16.70	50.80	40.90
13	21,117	52.90	BDL	14.20	113.30	10.50	2.00	35.30	8.70	57.70	47.50
14	19,413	43.40	1.60	14.30	113.00	9.00	2.10	34.40	26.10	58.30	43.20
15	28,658	77.40	BDL	19.80	162.20	10.20	1.10	47.40	24.60	94.40	55.40
16	31,003	91.60	1.40	102.50	379.70	15.60	BDL	76.80	87.00	165.50	73.50
17	8801	67.30	BDL	94.90	65.10	8.00	BDL	16.60	11.70	20.80	13.00
18	13,580	37.40	4.00	4.50	36.80	8.30	BDL	13.80	40.30	24.30	30.30
19	11,770	16.50	3.20	2.80	40.10	7.10	1.30	3.10	66.70	7.00	16.10
20	3906	40.70	BDL	6.20	111.10	9.10	BDL	7.80	93.90	20.50	30.70
21	2096	45.70	5.40	2.70	37.30	7.70	0.10	4.30	66.30	9.40	20.50
22	4488	47.90	BDL	1.30	50.10	7.10	4.10	3.50	4.40	5.30	19.00
23	8510	35.70	1.80	0.90	27.80	6.50	0.90	3.00	34.00	6.60	16.20
24	31,389	38.20	5.00	5.70	52.60	9.60	0.90	13.00	13.70	19.70	30.40
25	30,114	35.50	0.40	3.10	27.60	7.80	1.00	9.80	12.30	12.00	24.30
26	2773	67.30	0.60	113.10	38.60	6.70	3.10	2.30	5.30	2.40	2.90
27	3948	43.20	BDL	0.70	32.00	6.80	3.30	4.20	3.20	9.50	16.90
28	3985	65.10	BDL	66.50	50.10	7.40	3.90	5.50	3.50	4.40	1.80
29	2085	38.20	1.80	0.60	36.80	7.40	1.60	3.50	26.30	1.50	16.30
30	969	69.80	4.20	1.50	81.60	7.60	2.40	4.10	76.40	3.80	17.20
31	4985	53.70	BDL	0.90	11.50	7.10	0.50	2.30	14.30	5.50	16.30
32	3302	52.20	1.20	0.10	43.00	7.10	1.40	4.20	5.10	10.30	19.50
33	2831	73.70	0.20	120.80	40.20	7.00	2.10	3.70	BDL	5.30	BDL
34	1831	88.10	3.70	109.40	42.50	6.80	4.80	5.00	BDL	1.20	BDL
35	1609	66.40	1.50	53.60	37.80	7.00	1.80	4.00	BDL	0.10	BDL
36	9293	51.90	4.00	2.40	59.60	9.10	1.80	3.40	16.60	7.50	19.00
37	11,151	41.30	0.80	2.10	26.20	6.90	2.90	4.20	7.00	7.10	20.30
38	5414	25.30	1.80	0.20	31.40	6.90	0.80	3.60	48.50	6.20	16.00
39	1974	55.10	BDL	97.70	48.80	7.30	2.80	4.10	BDL	BDL	2.50
40	2429	33.70	4.30	BDL	33.30	9.80	0.90	3.30	18.80	1.60	17.40
Min.	969	16.50	BDL	BDL	11.50	6.50	BDL	2.30	BDL	BDL	BDL
Max.	31,389	91.60	5.40	120.80	379.70	15.60	4.80	76.80	93.90	165.50	73.50
Average	9655	51.80	1.67	26.05	63.07	8.05	1.55	11.30	25.96	18.85	21.30
UCC	80,400	1.50	0.098	10.00	35.00	25.00	1.50	20.00	20.00	60.00	71.00

BDL: Below detection limit, UCC: Upper Continental Crust [51].

**Table 2:** Minimum, maximum, and average values of EF and I_geo_ in Nasser Lake sediments.

		As	Cd	Co	Cr	Cu	Mo	Ni	Pb	V	Zn
EF	Min.	63.19	0.00	0.00	2.11	0.83	0.00	1.06	0.00	0.00	0.00
	Max.	3862.92	3557.74	480.33	200.38	25.24	140.50	20.56	317.11	7.15	20.11
	Average	725.64	431.51	56.24	31.84	6.12	24.51	5.83	29.59	2.43	4.24
	Min.	2.87	0.00	−7.23	−2.19	−2.53	−4.49	−3.71	−3.23	−9.81	−5.89
I_geo_	Max.	5.35	5.20	3.01	2.85	−1.27	1.09	1.36	1.65	0.88	−0.54
	Average	4.45	2.53	−1.51	−0.06	−2.24	−0.57	−2.13	−0.60	−3.29	−2.32

**Table 3:** Minimum, maximum, and average values of P_n_, PLI, and PERI in Nasser Lake sediments.

	P_n_	PLI	PERI
Min.	2.71	0.42	298.34
Max.	3.95	4.43	1981.13
Average.	3.32	1.10	887.08

**Table 4:** The correlation coefficient of measured PTEs and other components in Nasser Lake sediments.

	OM	Al_2_O_3_	Fe_2_O_3_	MnO	TiO_2_	CaO	As	Cd	Co	Cr	Cu	Mo	Ni	Pb	V	Zn
Mud	0.39	0.51	−0.05	0.05	0.10	−0.30	−0.35	0.00	−0.24	−0.15	−0.05	−0.10	−0.15	−0.03	−0.13	0.00
OM		0.75	0.64	0.44	0.70	0.17	0.09	−0.26	−0.02	0.36	0.48	−0.17	0.57	−0.03	0.55	0.65
Al_2_O_3_			0.78	0.58	0.85	0.17	0.12	−0.13	0.02	0.51	0.66	−0.31	0.72	0.07	0.70	0.73
Fe_2_O_3_				0.77	0.94	0.50	0.22	−0.20	0.04	0.53	0.69	−0.30	0.85	0.06	0.80	0.84
MnO					0.73	0.42	−0.03	0.02	−0.07	0.26	0.38	−0.32	0.53	0.14	0.50	0.56
TiO_2_						0.44	0.23	−0.22	0.06	0.67	0.79	−0.37	0.89	0.20	0.87	0.89
CaO							0.05	−0.03	−0.24	0.25	0.33	−0.18	0.40	0.08	0.43	0.53
As								−0.26	0.75	0.46	0.35	0.22	0.47	−0.17	0.45	0.11
Cd									−0.30	−0.10	0.01	−0.19	−0.21	0.36	−0.19	−0.08
Co										0.29	0.19	0.23	0.27	−0.19	0.24	−0.18
Cr											0.87	−0.26	0.87	0.48	0.89	0.74
Cu												−0.38	0.88	0.35	0.89	0.83
Mo													−0.26	−0.54	−0.28	−0.40
Ni														0.21	0.99	0.88
Pb															0.27	0.34
V																0.88
		Very Weak			Weak			Moderate			Strong			Very Strong		

**Table 5:** Measured radionuclides activity concentrations and radiation hazard indices values in Nasser Lake sediments.

Sample No.	^238^U (Bq kg^−1^)	^232^Th (Bq kg^−1^)	^226^Ra (Bq kg^−1^)	^40^K (Bq kg^−1^)	eTh/eU	eTh/K	eU/Ra	Ra_eq_	H_ex_	D	AEDE	ELCR × 10^−3^
1	49.60	48.48	11.10	65.73	3.00	57.14	4.00	85.41	0.33	54.94	0.07	0.24
2	8.31	36.36	11.10	21.91	13.43	128.57	0.67	64.73	0.17	26.71	0.03	0.11
3	37.20	44.44	22.20	15.65	3.67	220.00	1.50	86.89	0.28	44.68	0.05	0.19
4	74.40	8.08	7.55	184.67	0.33	3.39	8.82	33.30	0.27	46.95	0.06	0.20
5	37.20	8.08	11.10	56.34	0.67	11.11	3.00	26.98	0.14	24.42	0.03	0.10
6	12.40	8.08	22.20	140.85	2.00	4.44	0.50	44.58	0.09	16.48	0.02	0.07
7	37.20	12.12	22.20	50.08	1.00	18.75	1.50	43.37	0.16	26.60	0.03	0.11
8	74.40	4.04	11.10	278.57	0.17	1.12	6.00	38.30	0.27	48.43	0.06	0.21
9	8.18	8.08	11.10	25.04	3.03	25.00	0.66	24.57	0.06	9.71	0.01	0.04
10	10.91	8.08	9.77	115.81	2.27	5.41	1.00	30.22	0.08	14.75	0.02	0.06
11	12.40	20.20	6.22	300.48	5.00	5.21	1.79	58.19	0.17	30.46	0.04	0.13
12	12.40	20.20	11.10	84.51	5.00	18.52	1.00	46.46	0.13	21.45	0.03	0.09
13	24.80	16.16	9.55	162.76	2.00	7.69	2.33	45.15	0.16	28.01	0.03	0.12
14	49.60	8.08	33.30	184.67	0.50	3.39	1.33	59.05	0.20	35.50	0.04	0.15
15	12.40	28.28	22.20	93.90	7.00	23.33	0.50	69.82	0.16	26.73	0.03	0.11
16	62.00	52.52	44.40	18.78	2.60	216.67	1.25	120.87	0.37	61.15	0.07	0.26
17	24.80	20.20	22.20	153.37	2.50	10.20	1.00	62.85	0.18	30.05	0.04	0.13
18	6.94	44.44	22.20	81.38	19.64	42.31	0.28	91.95	0.21	33.44	0.04	0.14
19	24.80	16.16	22.20	6.26	2.00	200.00	1.00	45.77	0.13	21.48	0.03	0.09
20	74.40	32.32	22.20	43.82	1.33	57.14	3.00	71.74	0.33	55.72	0.07	0.24
21	10.29	20.20	22.20	81.38	6.02	19.23	0.42	57.32	0.12	20.35	0.02	0.09
22	37.20	20.20	11.10	53.21	1.67	29.41	3.00	44.05	0.19	31.61	0.04	0.14
23	12.40	8.08	11.10	165.89	2.00	3.77	1.00	35.40	0.10	17.53	0.02	0.08
24	62.00	16.16	11.10	3.13	0.80	400.00	5.00	34.43	0.23	38.54	0.05	0.17
25	136.40	32.32	9.77	13.77	0.73	181.82	12.50	57.00	0.50	83.11	0.10	0.36
26	24.80	8.08	11.10	15.65	1.00	40.00	2.00	23.85	0.10	16.99	0.02	0.07
27	24.80	12.12	11.10	131.46	1.50	7.14	2.00	38.53	0.14	24.26	0.03	0.10
28	62.00	16.16	11.10	103.29	0.80	12.12	5.00	42.13	0.25	42.71	0.05	0.18
29	8.06	8.08	11.10	40.69	3.08	15.38	0.65	25.77	0.06	10.30	0.01	0.04
30	5.58	8.08	11.10	143.98	4.44	4.35	0.45	33.72	0.08	13.46	0.02	0.06
31	6.08	12.12	7.33	137.72	6.12	6.82	0.74	35.23	0.09	15.87	0.02	0.07
32	8.18	12.12	11.10	172.15	4.55	5.45	0.66	41.66	0.10	18.28	0.02	0.08
33	62.00	6.26	11.10	25.04	0.31	19.38	5.00	21.97	0.20	33.47	0.04	0.14
34	8.18	8.08	8.88	169.02	3.03	3.70	0.83	33.42	0.09	15.71	0.02	0.07
35	9.55	4.04	11.10	68.86	1.30	4.55	0.77	22.17	0.06	9.72	0.01	0.04
36	24.80	8.08	11.10	18.78	1.00	33.33	2.00	24.09	0.10	17.12	0.02	0.07
37	49.60	12.12	11.10	187.80	0.75	5.00	4.00	42.86	0.22	38.07	0.05	0.16
38	6.82	24.24	11.10	100.16	10.91	18.75	0.55	53.43	0.13	21.97	0.03	0.09
39	12.40	8.08	9.10	87.64	2.00	7.14	1.22	27.39	0.08	14.26	0.02	0.06
40	12.28	24.24	11.10	9.39	6.06	200.00	0.99	46.45	0.13	20.70	0.03	0.09
Min.	5.58	4.04	6.22	3.13	0.17	1.12	0.28	21.97	0.06	9.71	0.01	0.04
Max.	136.40	52.52	44.40	300.48	19.64	400.00	12.50	120.87	0.50	83.11	0.10	0.36
Average	30.94	17.83	14.47	95.34	3.38	51.92	2.25	47.28	0.17	29.04	0.04	0.12
World average	33	45	32	412								

**Table 6:** The correlation coefficient of measured natural radionuclides and other components in Nasser Lake sediments.

	OM	Al_2_O_3_	Fe_2_O_3_	MnO	TiO_2_	CaO	^238^U	^232^Th	^40^K
Mud	0.39	0.51	−0.05	0.05	0.10	−0.30	0.44	0.41	−0.38
OM		0.75	0.64	0.44	0.70	0.17	0.31	0.57	0.10
Al_2_O_3_			0.78	0.58	0.85	0.17	0.37	0.57	−0.16
Fe_2_O_3_				0.77	0.94	0.50	0.07	0.42	0.13
MnO					0.73	0.42	0.02	0.38	−0.01
TiO_2_						0.44	0.22	0.64	−0.03
CaO							−0.22	0.16	0.20
^238^U								0.20	−0.11
^232^Th									−0.34
	very weak		weak		moderate		strong		very strong

## Data Availability

Data is contained within the article or Supplementary Materials.

## Supplementary Materials

The following supporting information can be downloaded at: https://www.mdpi.com/article/10.3390/toxics13090745/s1, Table S1: An overview of the equations used to determine the single and integrated metal contamination indices. Table S2: An overview of the equations used to determine the radiation hazard indices. Table S3: Percentages of the size fractions and textural nomenclature of the studied sediments. Table S4: Grain size parameters of the studied sediments. Table S5: Heavy index, heavy mineral relative abundances (%), and ZTR index in the studied sediments. Table S6: Concentrations of major oxides (%) in Nasser Lake sediments. Figure S1: Probability cumulative curves of the studied sediments. Figure S2: Boxplots of the studied sediments’ grain size parameters. Figure S3: SEM micrographs and chemical analysis results of precious minerals detected in mud sized fractions in the studied sediments: (a) and (b) show gold grain and its chemical composition, respectively, (c) and (e) display silver mineral grains, while (d) and (f) represent their chemical compositions, respectively. The oxygen percentage was excluded from the (b) and (f) chemical composition tables to clarify the other element percentages. Figure S4: SEM micrographs and chemical analysis results of radioactive and REEs bearing minerals detected in mud-sized fraction in the studied sediments: (a) and (b) show monazite grain and its chemical composition, respectively; (c) and (d) display brannerite grain and its chemical composition, respectively; (e) and (f) display allanite grain and its chemical composition, respectively; (g) and (h) show thorite grain and its chemical composition, respectively. The oxygen percentage was excluded from the (d) and (h) chemical composition tables to clarify the other elements percentages. Figure S5: SEM micrographs and chemical analysis results of radioactive and REEs bearing minerals detected in mud sized fraction in the studied sediments: (a) and (b) show uranophane grain and its chemical composition, respectively; (c) and (d) display cerianite grain and its chemical composition, respectively; (e) and (f) display silicate mineral grain containing REEs and its chemical composition, respectively; (g) and (h) show carbonate mineral grain containing REEs and its chemical composition, respectively. The oxygen percentage was excluded from the (b) chemical composition table to clarify the other elements percentages.
