# Supplementary material for: Potentially Toxic Elements and Natural Radioactivity in Nasser Lake Sediments: Environmental Risks in a Key Egyptian Freshwater Lake
Source: Toxics. 2025 Aug 31;13(9):745. doi: 10.3390/toxics13090745 (PMC12473842; doi:10.3390/toxics13090745)
Supplement: Supplementary file 1 [file toxics-13-00745-s001.zip › toxics-3834016-supplementary.pdf]

toxics

MDPI

SUPPORTING INFORMATION

Potentially Toxic Elements and Natural Radioactivity in Nasser Lake Sediments: Environmental Risks in a Key Egyptian Freshwater Lake

Total Pages: 11 including cover page
Total Tables: 6
Total Figures: 5

Table S1. An overview of the equations used to determine the single and integrated metals contamination indices.

Pollution Index	Categories	Reference
Enrichment Factor<br>EF= $(E/Al)_{sample}/(E/Al)_{crust}$	EF < 2: no enrichment<br>EF = 2–5: moderate enrichment<br>EF = 5–20: significant enrichment<br>EF = 20–40: very high enrichment<br>EF > 40: extremely high enrichment	[45]
Geo-accumulation index (I_{geo})<br>$I_{geo} = \log_2 (C_n / 1.5B_n)$	I_{geo} < 0: practically uncontaminated<br>I_{geo} = 0–1: uncontaminated to moderately contaminated<br>I_{geo} = 1–2: moderately contaminated<br>I_{geo} = 2–3: moderately to heavily contaminated<br>I_{geo}= 3–4: heavily contaminated<br>I_{geo} = 4–5: heavily to extremely contaminated<br>I_{geo} > 5: extremely contaminated	[46,47]
Improved Nemerow's Pollution Index (Pn)<br>$P_n = \sqrt{(I_{geomax}^2 + I_{geoave}^2)/2}$	P_n = 0–0.5: practically uncontaminated<br>P_n = 0.5–1: uncontaminated to moderately contaminated<br>P_n = 1–2: moderately contaminated<br>P_n = 2–3: moderately to heavily contaminated<br>P_n = 3–4: heavily contaminated<br>P_n = 4–5: heavily to extremely contaminated<br>P_n > 5: extremely contaminated	[48]
Pollution Load Index (PLI)<br>$PLI = (C_f^1 * C_f^2 * C_f^3 * \ldots * C_f^n)^{\frac{1}{n}}$<br>$C_f = C_{metal} / C_{background}$	PLI < 1: unpolluted<br>PLI = 1–2: moderately polluted<br>PLI = 2–10: strongly polluted<br>PLI > 10: extremely polluted	[49,50]

Potential Ecological Risk Index (PERI) $$PERI = \sum_{i}^{n} E_r^i$$ $$E_r^i = T_r^i * C_f^i$$	PERI ≤ 50: low risk<br>50 < PERI ≤ 100: moderate risk<br>100 < PERI ≤ 150: high risk<br>150 < PERI ≤ 200: very high risk<br>PERI > 200: extreme risk	[48,49]

where $(E/Al)_{sample}$ and $(E/Al)_{crust}$ represent the ratio of the measured element E and Al concentrations in sediment samples and the upper continental crust, respectively. C_n indicates the observed concentration of PTE n in a soil sample, while B_n indicates the concentration of PTE n in the upper continental crust. I_{geomax} is the maximum value of I_{geo}, while I_{geoave} represents the arithmetic mean value of I_{geo}. n refers to the number of elements under study and C_f^n denotes the contamination factor for the nth element. C_{metal} means the element's concentration in a sediment sample, while $C_{Background}$ denotes to the element's concentration in the upper continental crust. E_r^i represent potential ecological risk for i^{th} PTE, while T_r^i is toxic response factor of ith PTE and C_f^i refers to the contamination factor. Toxic response factors for As, Cd, Co, Cr, Cu, Mo, Ni, Pb, V, and Zn are 10, 30, 5, 2, 5, 2, 5, 5, 2, and 1, respectively [48,49,72].

Table S2. An overview of the equations used to determine the radiation hazard indices.

Radiation Indices	Units	Safety level	Reference
Radium Equivalent Activity Index (Ra_{eq}) $Ra_{eq} = C_{Ra} + 1.43\ C_{Th} + 0.077\ C_K$	Bq kg^{-1}	< 370 Bq kg^{-1}	[52,53]
External Radiation Hazard Index (H_{ex}) $H_{ex} = \frac{C_{Ra}}{370} + \frac{C_{Th}}{259} + \frac{C_K}{4810}$	–	< 1	[53,54]
Absorbed Dose Rate (D) $D = 0.462\ C_{Ra} + 0.604\ C_{Th} + 0.0417\ C_K$	nGy h^{-1}	< 57 nGy h^{-1}	[53]
Annual Effective Dose Equivalent (AEDE) $AEDE = D \times T \times DCF \times F_O \times 10^{-6}$	mSv yr^{-1}	< 0.07 mSv yr^{-1}	[53]
Excess Lifetime Cancer Risk (ELCR) $ELCR = AEDE \times DL \times RF$	–	< 0.29 $\times 10^{-3}$	[55,56,57]

where C_{Ra}, C_{Th}, and C_K refer to ^{226}Ra, ^{232}Th, and ^{40}K in (Bq kg^{-1}) activity concentrations, respectively. T is the time, which is 8760 h y^{-1}, while DCF represents the dose conversion factor of 0.7 $SvGy^{-1}$ and F_o refers to the outdoor occupancy factor of 0.2 for residents [53]. DL refers to the lifespan duration (70 years), while RF denotes the cancer risk factor utilized for the public of 0.05 Sv^{-1} [55].

Table S3. Percentages of the size fractions and textural nomenclature of the studied sediments.

Sample No.		Sand%						Nomenclature	Organic Matter%
	Gravel%	V. Coarse	Coarse	Medium	Fine	V. Fine	Total	Mud%		
1	0.00	0.00	23.28	37.93	18.12	12.05	91.38	8.62	Sand	1.99
2	0.00	0.00	14.84	34.77	27.67	14.31	91.59	8.41	Sand	1.01
3	0.00	0.02	11.00	35.85	28.26	14.26	89.40	10.60	Muddy Sand	0.99
4	0.00	0.00	0.89	72.67	20.21	3.47	97.24	2.76	Sand	0.53
5	0.00	0.00	1.52	34.64	51.74	10.78	98.68	1.32	Sand	1.02
6	0.00	0.00	0.55	27.29	56.21	12.45	96.50	3.50	Sand	1.03
7	0.00	0.02	0.22	13.09	61.99	20.32	95.65	4.35	Sand	0.52
8	0.00	0.00	9.90	62.20	23.72	3.90	99.72	0.28	Sand	1.01
9	0.00	0.00	0.87	42.19	47.01	9.77	99.84	0.16	Sand	0.53
10	0.00	0.00	0.50	26.91	56.26	14.30	97.97	2.03	Sand	0.99
11	0.00	0.00	1.65	9.15	75.85	12.66	99.31	0.69	Sand	1.58
12	0.00	0.00	1.33	6.80	75.75	16.06	99.94	0.06	Sand	1.04
13	0.00	0.00	1.36	7.91	77.17	11.36	97.81	2.19	Sand	1.47
14	0.00	0.00	0.72	11.31	79.80	7.76	99.59	0.41	Sand	1.02
15	0.00	0.00	0.02	1.11	86.66	11.10	98.89	1.11	Sand	1.52
16	0.00	0.00	0.25	1.27	85.45	11.08	98.05	1.95	Sand	1.55
17	0.02	0.16	0.22	33.86	60.61	3.67	98.53	1.45	Sand	1.00
18	0.02	0.12	0.14	25.49	64.61	7.68	98.03	1.94	Sand	1.00
19	0.00	0.00	4.32	46.02	30.02	12.09	92.45	7.55	Sand	0.52
20	0.00	0.00	2.06	12.28	55.86	27.88	98.08	1.92	Sand	0.50
21	0.00	0.00	1.10	40.00	47.57	10.29	98.96	1.04	Sand	0.52
22	0.00	0.00	1.07	29.69	46.64	19.21	96.61	3.39	Sand	1.03
23	0.00	0.00	0.65	50.14	29.74	15.71	96.24	3.76	Sand	0.51
24	0.00	0.00	2.58	56.53	25.09	3.90	88.10	11.90	Muddy Sand	1.51
25	0.00	0.00	2.71	61.80	21.35	2.55	88.41	11.59	Muddy Sand	1.50
26	0.10	0.16	3.21	65.74	26.11	3.62	98.84	1.06	Sand	0.51
27	0.00	0.00	0.98	43.49	41.91	10.35	96.73	3.27	Sand	0.50
28	0.00	0.02	1.66	38.29	45.47	11.55	96.99	3.01	Sand	0.51
29	0.00	0.00	2.82	59.53	33.06	3.32	98.73	1.27	Sand	0.51
30	0.00	0.02	1.09	63.55	31.25	3.76	99.68	0.32	Sand	0.50
31	0.00	0.00	1.25	39.67	44.41	11.99	97.32	2.68	Sand	0.53
32	0.00	0.00	4.41	39.15	36.75	18.87	99.19	0.81	Sand	0.52
33	0.00	0.20	11.80	48.87	29.94	7.71	98.53	1.47	Sand	0.53
34	0.02	0.02	20.79	51.35	21.88	5.41	99.46	0.52	Sand	0.49
35	0.00	0.00	0.02	8.11	61.07	30.26	99.47	0.53	Sand	0.00
36	0.00	0.00	8.93	49.85	22.55	13.64	94.97	5.03	Sand	0.51
37	0.00	0.00	5.92	38.80	30.84	16.83	92.39	7.61	Sand	1.06
38	0.00	0.00	19.60	43.55	23.02	10.38	96.55	3.45	Sand	1.02
39	0.00	0.02	20.17	65.68	13.21	0.34	99.44	0.56	Sand	0.51
40	0.00	0.00	1.31	33.48	57.39	6.57	98.75	1.25	Sand	0.50
Min.	0.00	0.00	0.02	1.11	13.21	0.34	88.10	0.06		0.00
Max.	0.10	0.20	23.28	72.67	86.66	30.26	99.94	11.90		1.99
Average	0.00	0.02	4.69	36.75	44.31	11.08	96.85	3.15		0.85

Table S4. Grain size parameters of the studied sediments.

Sample No.	Mean Size (Mz)		Standard deviation (σ_I)		Skewness (SK_I)		Kurtosis (KG)	
1	1.93	Medium sand	1.25	Poorly sorted	0.21	Fine skewed	0.97	Mesokurtic
2	2.15	Fine sand	1.14	Poorly sorted	0.15	Fine skewed	0.97	Mesokurtic
3	2.29	Fine sand	1.09	Poorly sorted	0.23	Fine skewed	0.89	Platykurtic
4	1.89	Medium sand	0.55	Moderately well sorted	0.38	Strongly fine skewed	1.74	Very leptokurtic
5	2.26	Fine sand	0.64	Moderately well sorted	0.09	Nearly symmetrical	1.04	Mesokurtic
6	2.39	Fine sand	0.66	Moderately well sorted	0.13	Fine skewed	1.14	Leptokurtic
7	2.66	Fine sand	0.65	Moderately well sorted	0.14	Fine skewed	1.23	Leptokurtic
8	1.73	Medium sand	0.62	Moderately well sorted	0.14	Fine skewed	1.16	Leptokurtic
9	2.18	Fine sand	0.58	Moderately well sorted	0.17	Fine skewed	0.96	Mesokurtic
10	2.40	Fine sand	0.63	Moderately well sorted	0.10	Nearly symmetrical	1.05	Mesokurtic
11	2.52	Fine sand	0.49	Well sorted	-0.04	Nearly symmetrical	1.32	Leptokurtic
12	2.59	Fine sand	0.45	Well sorted	-0.06	Nearly symmetrical	1.15	Leptokurtic
13	2.55	Fine sand	0.50	Well sorted	0.04	Nearly symmetrical	1.42	Leptokurtic
14	2.46	Fine sand	0.43	Well sorted	-0.02	Nearly symmetrical	1.24	Leptokurtic
15	2.66	Fine sand	0.33	Very well sorted	0.12	Fine skewed	1.30	Leptokurtic
16	2.66	Fine sand	0.36	Well sorted	0.13	Fine skewed	1.39	Leptokurtic
17	2.21	Fine sand	0.48	Well sorted	0.03	Nearly symmetrical	0.99	Mesokurtic
18	2.34	Fine sand	0.54	Moderately well sorted	0.09	Nearly symmetrical	1.14	Leptokurtic
19	2.22	Fine sand	0.91	Moderately sorted	0.37	Strongly fine skewed	1.05	Mesokurtic
20	2.67	Fine sand	0.66	Moderately well sorted	-0.04	Nearly symmetrical	1.11	Mesokurtic
21	2.21	Fine sand	0.62	Moderately well sorted	0.16	Fine skewed	1.01	Mesokurtic
22	2.45	Fine sand	0.75	Moderately sorted	0.13	Fine skewed	0.97	Mesokurtic
23	2.25	Fine sand	0.77	Moderately sorted	0.48	Strongly fine skewed	0.98	Mesokurtic
24	2.11	Fine sand	0.84	Moderately sorted	0.46	Strongly fine skewed	1.23	Leptokurtic
25	2.05	Fine sand	0.81	Moderately sorted	0.50	Strongly fine skewed	1.34	Leptokurtic
26	1.86	Medium sand	0.55	Moderately well sorted	0.23	Fine skewed	1.18	Leptokurtic
27	2.22	Fine sand	0.69	Moderately well sorted	0.29	Fine skewed	1.11	Mesokurtic
28	2.25	Fine sand	0.71	Moderately well sorted	0.18	Fine skewed	1.07	Mesokurtic
29	1.92	Medium sand	0.55	Moderately well sorted	0.19	Fine skewed	1.07	Mesokurtic
30	1.93	Medium sand	0.50	Well sorted	0.25	Fine skewed	1.13	Leptokurtic
31	2.25	Fine sand	0.69	Moderately well sorted	0.21	Fine skewed	1.05	Mesokurtic
32	2.24	Fine sand	0.78	Moderately sorted	0.13	Fine skewed	0.87	Platykurtic
33	1.87	Medium sand	0.79	Moderately sorted	0.15	Fine skewed	1.05	Mesokurtic
34	1.64	Medium sand	0.74	Moderately sorted	0.19	Fine skewed	1.00	Mesokurtic
35	2.73	Fine sand	0.51	Moderately well sorted	-0.02	Nearly symmetrical	0.97	Mesokurtic
36	2.08	Fine sand	0.96	Moderately sorted	0.34	Strongly fine skewed	1.04	Mesokurtic
37	2.33	Fine sand	0.97	Moderately sorted	0.23	Fine skewed	0.93	Mesokurtic
38	1.83	Medium sand	1.02	Poorly sorted	0.19	Fine skewed	1.10	Mesokurtic
39	1.45	Medium sand	0.52	Moderately well sorted	0.09	Nearly symmetrical	1.00	Mesokurtic
40	2.22	Fine sand	0.57	Moderately well sorted	0.05	Nearly symmetrical	1.08	Mesokurtic
Min.	1.45	Medium sand	0.33	Very well sorted	-0.06	Nearly symmetrical	0.87	Platykurtic
Max.	2.73	Fine sand	1.25	Poorly sorted	0.50	Strongly fine skewed	1.74	Very leptokurtic
Average	2.22	Fine sand	0.68	Moderately well sorted	0.17	Fine skewed	1.11	Mesokurtic

Table S5. Heavy index, heavy mineral relative abundances (%), and ZTR index in the studied sediments.

Sample No.	HI	Op	Px	Amp	Zrn	Tur	Rt	Grt	Ep	Mon	Ky	Sph	Bt	Ms	Ap	Sta	Sil	ZTR
1	0.64	64.29	10.86	7.71	5.14	2.00	3.14	0.57	1.43	1.71	0.29	0.00	0.00	1.43	0.29	0.86	0.29	30.00
2	0.99	68.03	9.13	7.21	6.01	0.72	3.13	0.48	1.44	1.20	0.24	0.24	0.00	0.96	0.48	0.72	0.00	31.78
3	0.70	69.98	8.55	5.08	7.16	1.85	2.31	0.46	0.23	1.62	0.23	0.00	0.46	0.23	0.23	1.62	0.00	38.58
4	1.80	35.31	40.11	13.84	1.69	0.56	1.41	0.56	1.69	0.85	0.00	1.41	0.00	0.00	0.85	1.13	0.56	5.68
5	1.10	34.23	46.63	12.13	1.62	0.27	0.81	0.27	1.35	0.27	0.27	0.00	0.54	0.00	0.00	1.62	0.00	4.13
6	1.10	46.65	33.76	9.02	1.80	1.03	1.55	0.77	1.03	1.29	0.26	0.26	0.00	0.00	0.52	2.06	0.00	8.21
7	1.06	58.99	17.13	5.06	6.74	0.56	1.97	2.53	3.09	2.25	0.28	0.00	0.56	0.00	0.00	0.84	0.00	22.92
8	1.38	48.98	28.34	13.38	0.91	0.91	0.91	0.45	2.72	1.81	0.23	0.00	0.45	0.00	0.23	0.68	0.00	5.38
9	0.24	52.59	22.84	12.93	2.80	0.65	0.22	1.29	2.37	2.16	0.22	0.22	0.22	0.22	0.43	0.86	0.00	7.80
10	1.24	37.25	32.38	21.20	2.01	0.29	2.01	0.00	2.29	1.43	0.29	0.00	0.00	0.00	0.29	0.57	0.00	6.85
11	1.80	23.80	52.41	16.87	0.60	0.60	0.30	0.30	3.31	1.20	0.30	0.00	0.30	0.00	0.00	0.00	0.00	1.98
12	15.67	25.89	41.09	26.37	1.19	0.00	0.95	0.95	1.43	0.95	0.24	0.24	0.24	0.00	0.48	0.00	0.00	2.89
13	18.04	21.15	49.73	21.98	1.37	0.27	0.82	1.10	1.37	0.55	0.27	0.00	0.00	0.27	0.82	0.27	0.00	3.15
14	17.08	34.27	41.85	18.82	1.97	0.00	0.28	0.28	1.40	0.28	0.28	0.28	0.00	0.00	0.00	0.28	0.00	3.42
15	19.20	22.86	49.65	20.79	1.85	0.23	0.69	0.23	0.69	1.15	0.23	0.23	0.00	0.00	0.46	0.92	0.00	3.59
16	27.73	25.68	50.27	20.22	0.82	0.27	0.27	0.55	1.64	0.00	0.00	0.00	0.00	0.00	0.27	0.00	0.00	1.84
17	30.37	31.18	48.55	11.58	2.00	0.00	0.67	1.11	1.34	1.78	0.22	0.00	0.00	0.00	0.45	1.11	0.00	3.88
18	6.76	39.16	40.73	12.53	1.83	0.52	0.78	0.52	1.83	1.04	0.00	0.26	0.00	0.00	0.52	0.26	0.00	5.15
19	9.00	75.55	7.42	5.02	6.11	1.31	1.75	0.22	0.00	1.09	0.00	0.00	0.00	0.00	0.44	1.09	0.00	37.50
20	0.30	78.43	4.48	1.96	8.12	1.96	2.24	0.56	0.28	0.56	0.28	0.00	0.00	0.00	0.28	0.84	0.00	57.14
21	1.50	76.10	4.40	0.82	7.14	1.92	5.22	0.55	0.27	2.47	0.27	0.27	0.00	0.00	0.00	0.55	0.00	59.77
22	0.80	76.32	5.92	2.19	7.24	0.88	3.07	0.44	0.66	1.97	0.22	0.44	0.00	0.00	0.00	0.66	0.00	47.22
23	0.91	71.25	5.00	5.00	6.00	1.25	2.75	1.50	0.00	5.00	0.25	0.00	0.00	0.00	1.00	1.00	0.00	34.78
24	0.74	79.06	7.35	4.45	2.90	1.78	1.11	0.45	0.45	0.45	0.00	0.00	0.00	0.00	0.45	1.56	0.00	27.66
25	0.24	81.64	4.96	5.46	2.73	0.99	1.99	0.00	0.50	0.99	0.25	0.00	0.00	0.00	0.00	0.50	0.00	31.08
26	0.18	77.87	8.47	6.01	2.19	0.82	1.91	0.00	0.55	1.64	0.00	0.00	0.00	0.00	0.00	0.55	0.00	22.22
27	0.16	71.04	2.09	5.07	9.25	1.19	1.49	1.19	0.30	3.28	0.60	0.00	0.00	0.00	0.30	3.88	0.30	41.24
28	0.88	71.84	6.15	8.74	6.15	0.65	2.27	0.32	0.32	1.94	0.32	0.32	0.00	0.00	0.00	0.97	0.00	32.18
29	0.42	68.10	7.76	6.61	5.75	1.72	2.30	1.15	0.29	4.02	0.00	0.29	0.00	0.29	0.29	1.44	0.00	30.91
30	0.47	70.76	8.36	7.57	4.18	0.78	1.83	1.04	0.26	2.61	0.26	0.00	0.00	0.00	0.52	1.83	0.00	23.21
31	0.76	69.87	5.80	7.14	5.80	1.12	2.90	0.45	0.45	3.79	0.67	0.45	0.00	0.22	0.22	1.12	0.00	32.84
32	1.28	74.93	7.04	4.23	4.51	0.56	1.69	0.85	0.56	4.23	0.00	0.00	0.00	0.00	0.56	0.85	0.00	26.97
33	0.34	73.18	7.54	6.15	5.03	0.84	1.40	0.28	0.28	4.19	0.28	0.00	0.00	0.00	0.00	0.84	0.00	27.08
34	0.26	71.27	9.30	7.89	2.82	0.28	1.97	0.56	0.28	4.51	0.00	0.00	0.00	0.00	0.56	0.56	0.00	17.65
35	1.61	56.27	18.66	11.37	2.33	2.33	2.92	1.17	0.58	1.75	0.29	0.58	0.00	0.00	0.58	0.87	0.29	17.33
36	0.52	73.46	6.64	5.45	2.37	1.18	2.37	1.42	0.71	4.27	0.24	0.24	0.00	0.00	0.47	1.18	0.00	22.32
37	0.86	77.65	2.79	3.07	6.70	1.96	3.63	0.84	0.28	1.12	0.28	0.28	0.00	0.00	0.28	1.12	0.00	55.00
38	0.55	78.07	3.92	2.87	5.22	0.78	3.39	0.52	0.26	2.87	0.26	0.26	0.00	0.00	0.52	1.04	0.00	42.86
39	0.56	26.18	43.72	20.68	0.52	1.57	1.05	0.00	2.62	1.31	0.00	0.00	0.00	0.00	0.26	1.83	0.26	4.26
40	1.47	61.24	15.64	10.42	4.89	1.63	0.65	0.65	1.63	0.98	0.00	0.00	0.00	0.00	0.00	2.28	0.00	18.49
Min.	0.16	21.15	2.09	0.82	0.52	0.00	0.22	0.00	0.00	0.00	0.00	0.00	0.00	0.00	0.00	0.00	0.00	1.84
Max.	30.37	81.64	52.41	26.37	9.25	2.33	5.22	2.53	3.31	5.00	0.67	1.41	0.56	1.43	1.00	3.88	0.56	59.77
Average	4.27	57.51	20.44	9.87	3.89	0.96	1.80	0.66	1.05	1.91	0.21	0.16	0.07	0.09	0.33	1.01	0.04	22.42

HI=heavy index, Op=Opaue, Px=Pyroxene, Amp=Amphibole, Zrn=Zircon, Tur=Tourmaline, Rt=Rutile, Grt=Garnet, Ep=Epidote, Mon=Monazite, Ky=Kyanite, Sph=Sphene, Bt=Biotite, Ms=Muscovite, Ap=Apatite, Sta=Staurolite, Sil=Sillimanite.

Table S6. Concentrations of major oxides (%) in Nasser Lake sediments.

Sample No.	SiO_2	Al_2O_3	Fe_2O_3	MnO	CaO	Na_2O	MgO	TiO_2	K_2O	SO_3	P_2O_5
1	89.07	2.55	2.11	0.04	2.45	0.35	0.37	0.58	0.25	0.05	0.04
2	91.32	2.48	1.58	0.07	1.47	0.32	0.40	0.50	0.30	0.03	0.04
3	90.31	3.87	1.80	0.04	1.00	0.14	0.38	0.56	0.30	0.03	0.06
4	93.95	1.03	1.33	0.04	1.12	0.69	0.33	0.18	0.46	0.07	0.06
5	89.90	0.73	1.58	0.04	3.85	0.53	0.49	0.32	0.54	0.09	0.05
6	90.33	0.92	1.87	0.13	3.94	0.28	0.29	0.18	0.39	0.10	0.06
7	85.46	1.55	1.94	0.09	5.85	1.03	0.50	0.29	0.59	0.07	0.03
8	93.42	0.23	0.97	0.03	2.24	0.33	0.27	0.14	0.45	0.07	0.03
9	95.01	0.27	0.73	0.04	1.40	0.40	0.22	0.12	0.23	0.03	0.02
10	90.36	1.04	1.44	0.03	3.23	0.68	0.40	0.20	0.39	0.04	0.03
11	76.53	3.00	6.99	0.10	4.48	1.58	1.58	1.01	1.07	0.14	0.08
12	72.67	3.65	8.80	0.10	5.03	2.15	2.03	1.12	1.26	0.09	0.09
13	70.86	3.99	8.60	0.13	6.15	2.09	2.34	1.28	1.29	0.14	0.14
14	75.13	3.67	7.49	0.11	4.78	1.76	2.05	1.11	1.30	0.07	0.12
15	68.06	5.42	9.58	0.16	6.45	1.90	2.78	1.52	0.96	0.04	0.12
16	70.07	5.86	8.08	0.11	5.11	1.93	2.79	1.89	0.69	0.05	0.10
17	86.33	1.66	2.83	0.07	3.23	1.27	0.81	0.50	0.74	0.10	0.04
18	77.74	2.57	6.72	0.21	6.23	0.53	0.61	1.21	0.85	0.17	0.17
19	92.53	2.22	1.91	0.11	1.08	0.32	0.33	0.44	0.07	0.10	0.04
20	86.42	0.74	1.47	0.03	5.43	0.17	0.33	0.60	0.25	0.06	0.04
21	88.72	0.40	1.12	0.04	5.05	0.33	0.23	0.30	0.26	0.05	0.04
22	86.35	0.85	1.35	0.04	6.48	0.35	0.28	0.32	0.28	0.05	0.07
23	85.71	1.61	1.24	0.06	6.77	0.40	0.24	0.13	0.31	0.07	0.03
24	83.54	5.93	3.47	0.05	1.47	0.20	1.23	0.69	0.60	0.07	0.06
25	83.47	5.69	5.24	0.15	0.75	0.15	0.53	1.10	0.65	0.12	0.06
26	95.25	0.52	0.62	0.05	1.29	0.25	0.21	0.08	0.15	0.06	0.03
27	92.03	0.75	1.04	0.04	2.73	0.56	0.29	0.18	0.29	0.07	0.02
28	91.38	0.75	1.04	0.04	3.47	0.12	0.20	0.22	0.25	0.12	0.03
29	91.75	0.39	1.66	0.09	3.82	0.08	0.13	0.24	0.35	0.06	0.05
30	96.38	0.18	0.49	0.03	0.99	0.25	0.10	0.10	0.23	0.02	0.01
31	89.01	0.94	2.71	0.05	4.35	0.40	0.27	0.34	0.28	0.06	0.08
32	89.28	0.62	1.27	0.03	4.10	0.28	0.27	0.23	0.27	0.06	0.04
33	97.12	0.54	0.14	0.03	0.22	0.06	0.04	0.01	0.12	0.01	0.01
34	93.96	0.35	0.73	0.04	2.36	0.19	0.15	0.06	0.23	0.04	0.02
35	95.18	0.30	0.58	0.04	2.08	0.26	0.09	0.06	0.09	0.06	0.02
36	88.79	1.76	1.05	0.04	4.22	0.36	0.26	0.10	0.24	0.10	0.06
37	90.01	2.11	1.37	0.02	2.72	0.53	0.28	0.19	0.27	0.04	0.05
38	92.09	1.02	1.89	0.06	2.41	0.34	0.33	0.18	0.21	0.10	0.07
39	96.44	0.37	0.57	0.02	0.84	0.26	0.28	0.05	0.15	0.05	0.02
40	92.29	0.46	1.46	0.04	2.91	0.37	0.24	0.16	0.39	0.04	0.04
Min.	68.06	0.18	0.14	0.02	0.22	0.06	0.04	0.01	0.07	0.01	0.01
Max.	97.12	5.93	9.58	0.21	6.77	2.15	2.79	1.89	1.30	0.17	0.17
Average	87.61	1.82	2.67	0.07	3.34	0.60	0.62	0.46	0.45	0.07	0.05

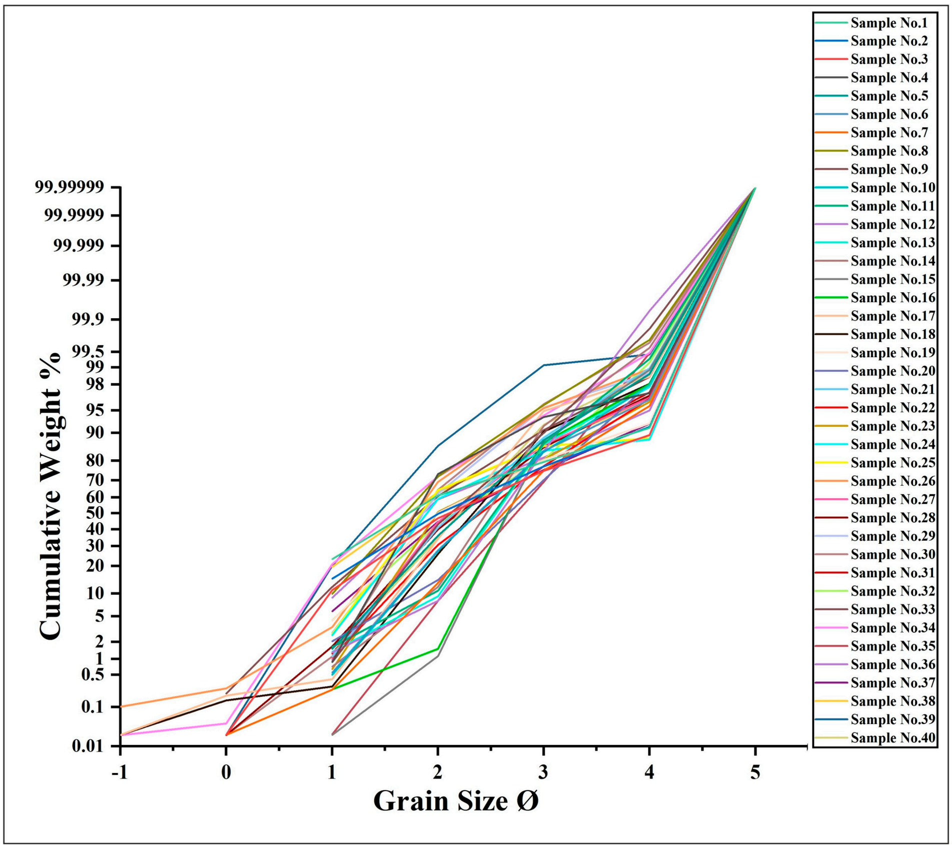

Figure S1. Probability cumulative curves of the studied sediments.

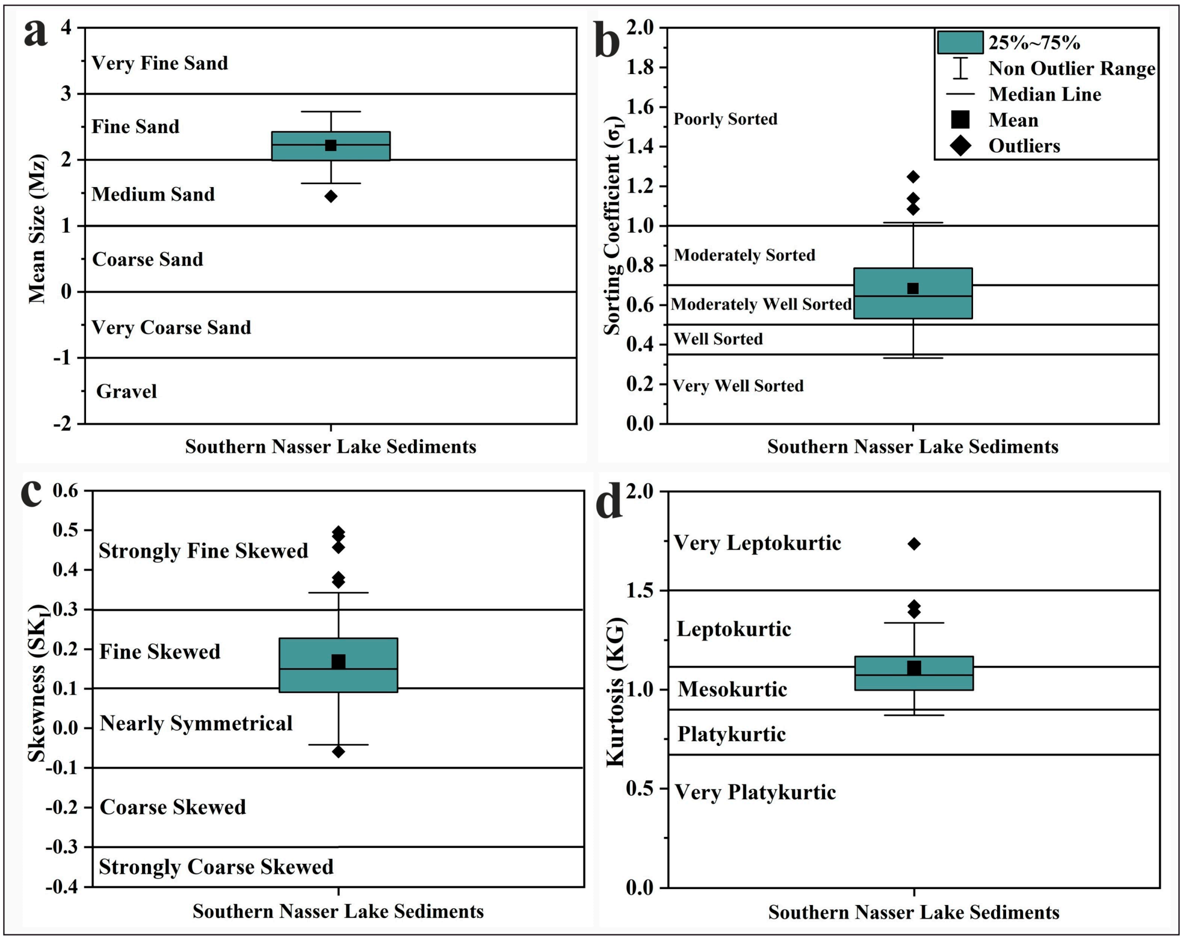

Figure S2. Boxplots of the studied sediments' grain size parameters.

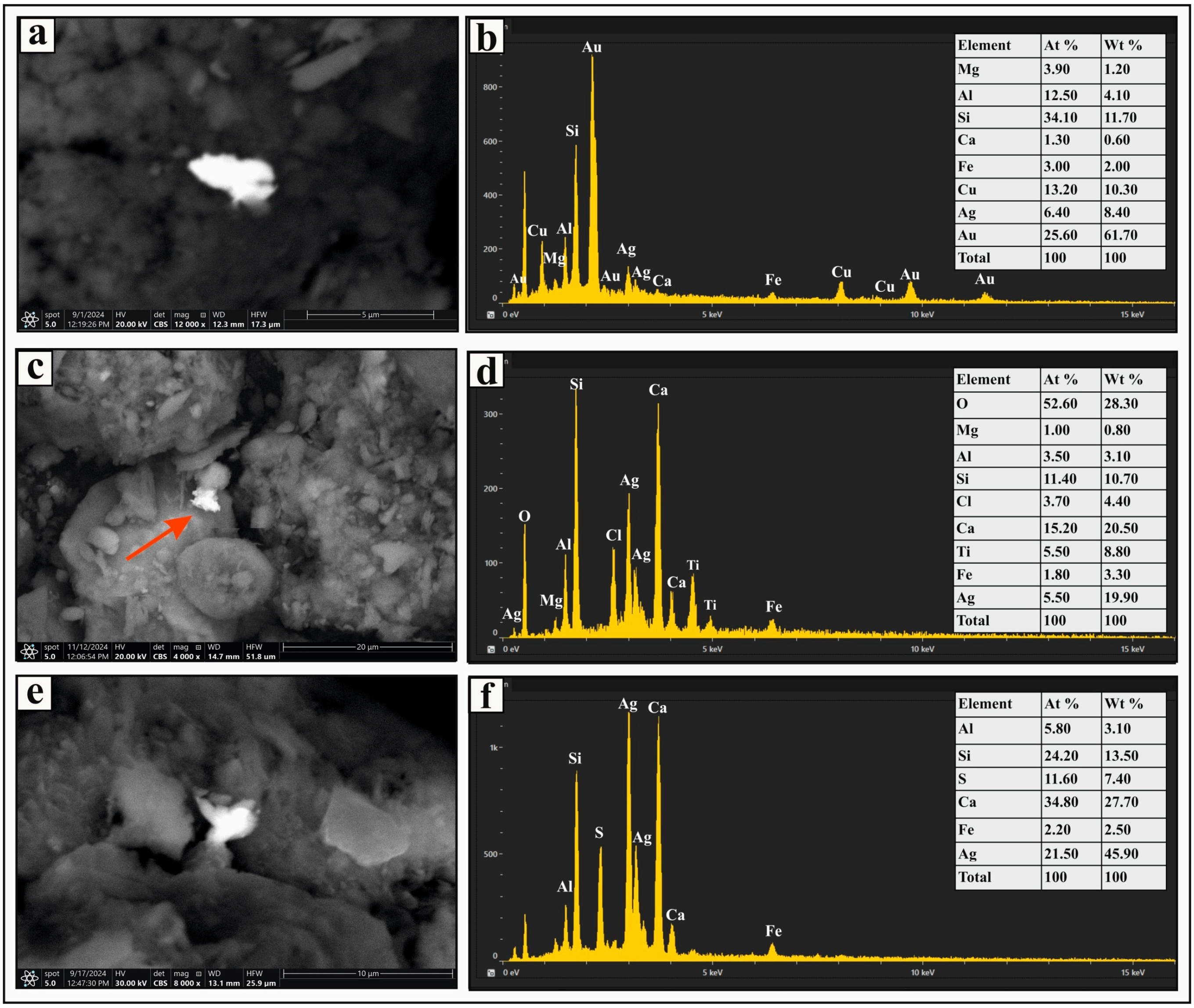

Figure S3. SEM micrographs and chemical analysis results of precious minerals detected in mud sized fractions in the studied sediments: (a) and (b) show gold grain and its chemical composition, respectively; (c) and (e) display silver minerals grains, while (d) and (f) represent their chemical compositions, respectively. The oxygen percentage was excluded from (b) and (f) chemical composition tables to clarify the other elements percentages.

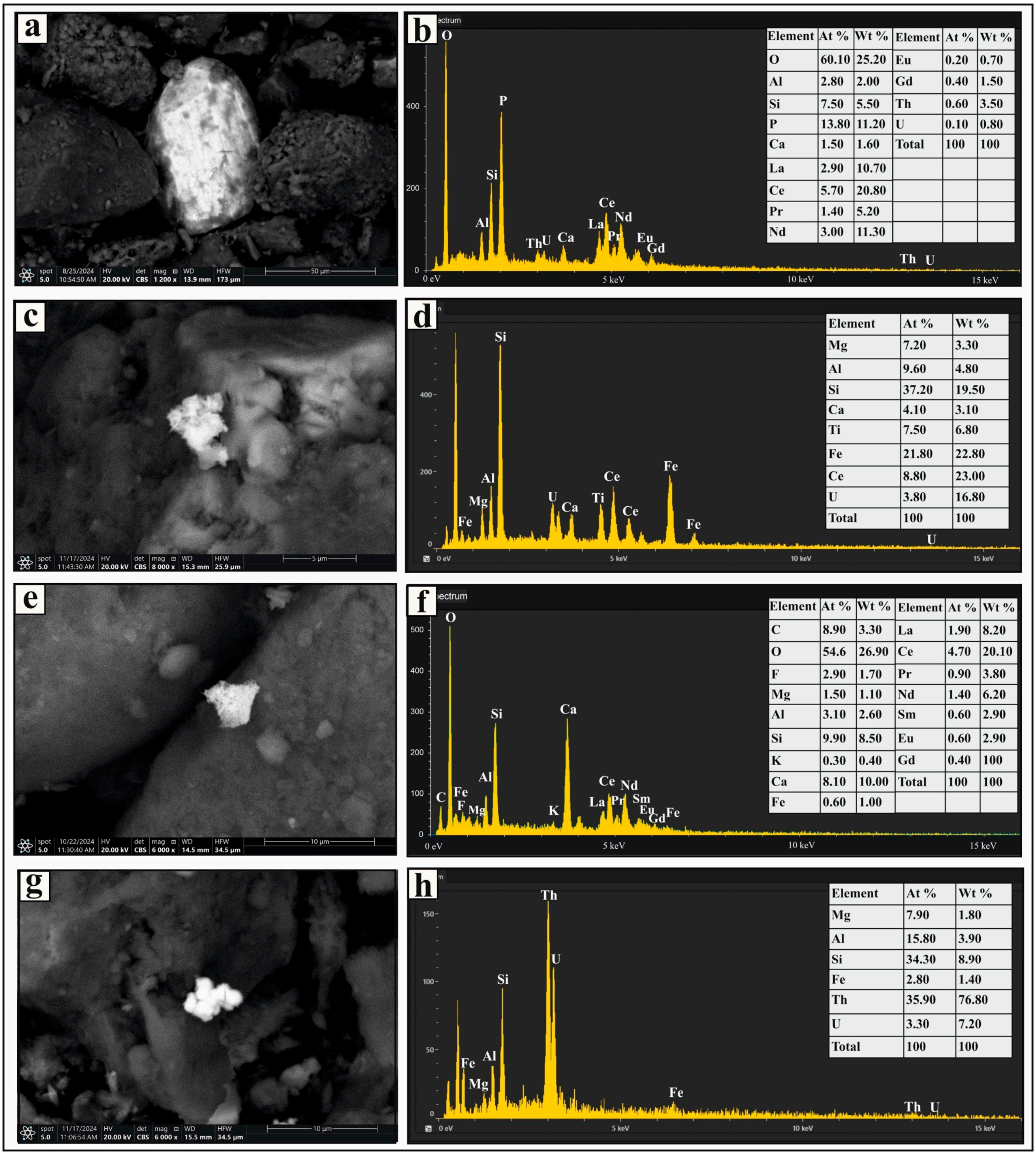

Figure S4. SEM micrographs and chemical analysis results of radioactive and REEs bearing minerals detected in mud sized fraction in the studied sediments: (a) and (b) show monazite grain and its chemical composition, respectively; (c) and (d) display brannerite grain and its chemical composition, respectively; (e) and (f) display allanite grain and its chemical composition, respectively; (g) and (h) show thorite grain and its chemical composition, respectively. The oxygen percentage was excluded from (d) and (h) chemical composition tables to clarify the other elements percentages.

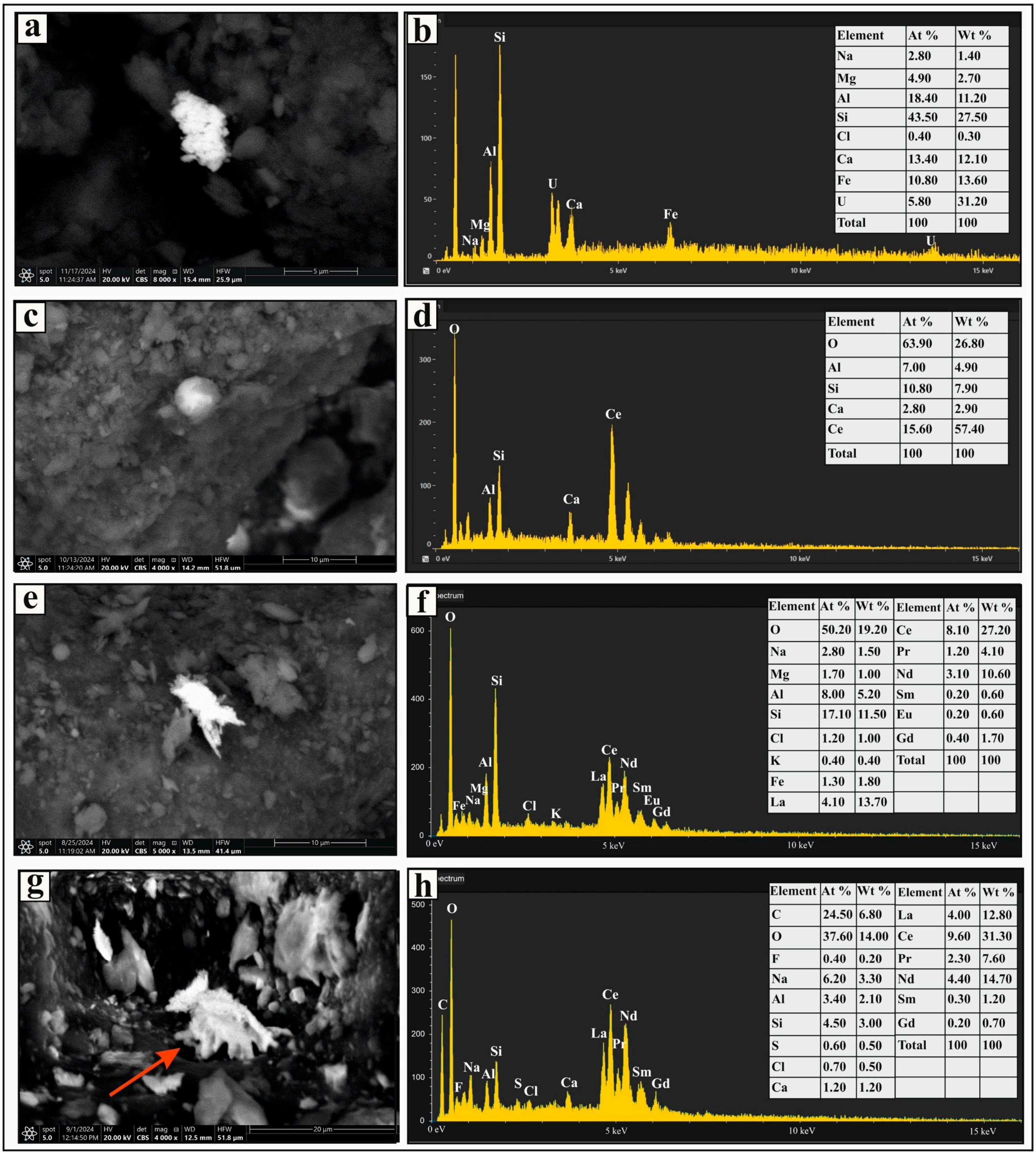

Figure S5. SEM micrographs and chemical analysis results of radioactive and REEs bearing minerals detected in mud sized fraction in the studied sediments: (a) and (b) show uranophane grain and its chemical composition, respectively; (c) and (d) display cerianite grain and its chemical composition, respectively; (e) and (f) display silicate mineral grain containing REEs and its chemical composition, respectively; (g) and (h) show carbonate mineral grain containing REEs and its chemical composition, respectively. The oxygen percentage was excluded from (b) chemical composition table to clarify the other elements percentages.
